# Mitochondrial ROS in Retinal Neurodegeneration: Thresholds, Quality Control Failure, and Precision Therapeutic Windows

**DOI:** 10.3390/biom16030445

**Published:** 2026-03-16

**Authors:** Snježana Kaštelan, Antonela Gverović Antunica, Suzana Konjevoda, Zora Tomić, Ana Sarić, Marjan Kulaš, Lorena Kulaš, Emina Kujundžić Begović, Samir Čanović, Petra Kovačević, Mira Ivanković

**Affiliations:** 1 Department of Ophthalmology, Clinical Hospital Dubrava, 10000 Zagreb, Croatia; 2 Fundamentals of Medical Skills, School of Medicine, University of Zagreb, 10000 Zagreb, Croatia; 3 Department of Ophthalmology, Dubrovnik General Hospital, 20000 Dubrovnik, Croatia; 4 Department of Ophthalmology, Zadar General Hospital, 23000 Zadar, Croatia; 5 Department of Health Studies, University of Zadar, 23000 Zadar, Croatia; 6 Health Centre of the Croatian, Department of Internal Affairs, 10000 Zagreb, Croatia; 7 School of Medicine, Catholic University of Croatia, 10000 Zagreb, Croatia; 8 Postgraduate School, University of Split School of Medicine, 21000 Split, Croatia; 9 Department of Obstetrics and Gynaecology, University Hospital Centre Zagreb, 10000 Zagreb, Croatia; 10 Eye Clinic, Clinical Center University of Sarajevo, Bolnička 25, 71000 Sarajevo, Bosnia and Herzegovina; 11 Department of Ophthalmology, University Hospital Centre Zagreb, 10000 Zagreb, Croatia; 12 Department of Neurology, Dubrovnik General Hospital, 20000 Dubrovnik, Croatia

**Keywords:** mitochondrial reactive oxygen species (mtROS), retinal neurodegeneration, mitochondrial quality control, mitophagy, retinal ganglion cells, reverse electron transport, redox signalling, mitochondria-targeted intervention, precision medicine

## Abstract

Mitochondrial reactive oxygen species (mtROS) play a dual role in retinal physiology, acting as essential redox signalling mediators under homeostatic conditions but driving oxidative damage and neurodegeneration once regulatory thresholds are exceeded. Owing to the exceptionally high energetic demands of retinal neurons and supporting cells, even subtle perturbations in mitochondrial redox balance can precipitate progressive retinal dysfunction. Increasing evidence indicates that retinal neurodegenerative diseases, including glaucoma, diabetic retinopathy (DR), age-related macular degeneration (AMD), and inherited optic neuropathies, are characterised not by uniform oxidative stress, but by disease- and stage-specific mtROS signatures shaped by mitochondrial quality control capacity. This review synthesises current insights into the sources, regulation, and signalling functions of mtROS in the retina, with particular emphasis on threshold-dependent redox transitions, reverse electron transport, and the progressive failure of mitochondrial quality control mechanisms, including mitophagy, mitochondrial dynamics, and redox-responsive transcriptional networks. The limitations of non-selective antioxidant strategies are critically examined, highlighting why indiscriminate ROS suppression has yielded limited clinical benefit. In contrast, emerging therapeutic approaches aimed at recalibrating mitochondrial redox homeostasis, rather than abolishing physiological signalling, are discussed in the context of disease stage, metabolic state, and mitochondrial competence. By integrating redox biology with mitochondrial quality control and precision medicine concepts, this review proposes a unifying framework in which retinal neurodegeneration is governed by regulated mtROS signalling and the progressive exhaustion of mitochondrial resilience. This model defines critical therapeutic windows for mitochondria-targeted intervention and provides a framework for biomarker-guided patient stratification.

## 1. Introduction

Ocular neurodegenerative diseases, including glaucoma, age-related macular degeneration (AMD), diabetic retinopathy (DR), and Leber’s hereditary optic neuropathy (LHON), represent a growing global health burden and a leading cause of irreversible vision loss [1]. Glaucoma affects more than 57 million individuals worldwide and is projected to exceed 111 million cases by 2040 [2]. AMD remains the predominant cause of central vision loss in the elderly [3], DR imposes a substantial burden on working-age populations [4], and LHON causes profound bilateral vision loss driven by pathogenic mitochondrial DNA (mtDNA) mutations that affect complex I of the electron transport chain (ETC) [5,6]. Despite major advances in disease characterisation, the translation of experimental insights into effective neuroprotective therapies remains limited, reflecting both the multifactorial nature of ocular neurodegeneration and pronounced interindividual variability in disease progression and treatment response, including differences in mitochondrial resilience and redox regulation [7].

Across these clinically distinct disorders, mitochondrial dysfunction has emerged as a unifying pathogenic denominator, linking metabolic stress, impaired redox signalling, and progressive neuronal vulnerability. Interventions aimed at enhancing mitochondrial resilience may therefore be pharmacological, such as mitochondria-targeted antioxidants, or system-level/behavioural, exemplified by environmental enrichment strategies that promote mitochondrial function and neuroprotection [8]. Such approaches bridge lifestyle and molecular mechanisms, highlighting the potential of non-pharmacological interventions to complement drug-based therapies.

The retina operates under exceptionally high energetic demand, with photoreceptors and retinal ganglion cells (RGCs) relying on sustained mitochondrial adenosine triphosphate (ATP) production to support visual signalling and axonal transport [9]. Retinal mitochondria are continuously exposed to oxidative pressure arising from intense metabolic flux and environmental stressors. Further mtDNA, lacking protective histones and possessing limited repair capacity, accumulates oxidative lesions that further amplify mitochondrial reactive oxygen species (mtROS) production [10,11,12,13,14].

In this context, mtROS occupy a central and inherently paradoxical role. Excessive mtROS drives mitochondrial membrane damage, mtDNA instability, inflammasome activation, and apoptotic signalling cascades that contribute directly to neuronal loss [15,16]. Conversely, basal mtROS are indispensable for adaptive redox signalling and stress resilience, regulating mitochondrial quality control processes such as mitophagy, antioxidant gene expression, and metabolic adaptation [17,18,19,20]. Increasing evidence indicates that retinal neurodegeneration reflects a progressive failure of mitochondrial quality control systems, thereby converting physiological mtROS signalling into pathological amplification. The inability to distinguish between adaptive and maladaptive mtROS signalling has contributed substantially to the translational gap in treating ocular neurodegenerative diseases. The dual nature of mtROS can be contextualised within the broader “oxygen paradox,” which reflects the fundamental tension between oxygen’s essential role in sustaining high-energy metabolism and its potential to generate damaging reactive species. The retina exemplifies this paradox; its high metabolic rate and oxygen tension render photoreceptors and RGCs particularly vulnerable to oxidative stress, illustrating why even physiological levels of mtROS must be tightly regulated [21].

Current therapeutic strategies largely address downstream manifestations of disease rather than early mitochondrial vulnerability and resilience. In glaucoma, intraocular pressure reduction slows disease progression but does not correct the intrinsic mitochondrial susceptibility of RGCs [1,7,22]. Anti-vascular endothelial growth factor (anti-VEGF) therapies stabilise neovascular complications in AMD yet fail to prevent early mitochondrial degeneration within the retinal pigment epithelium (RPE) [23,24]. Improved glycaemic control in diabetes reduces long-term complications but does not adequately resolve mitochondrial overload and oxidative stress in DR [25,26]. Even in LHON, where mitochondrial dysfunction is primary, available treatments only partially restore bioenergetic capacity and do not fully prevent neurodegeneration [6].

Important mechanistic uncertainties further hinder progress. It remains unclear why RGCs exhibit disproportionate sensitivity to bioenergetic deficits in glaucoma and LHON [27,28], why RPE mitochondria in AMD are particularly susceptible to lipid peroxidation and complement-driven inflammation [29,30,31], or how vascular and neuronal mitochondrial dysfunction converge to destabilise the neurovascular unit in DR [32,33]. The thresholds at which physiological mtROS signalling shifts into pathological amplification, particularly via reverse electron transport (RET) and impaired mitophagy, remain insufficiently defined [34,35,36].

Recent advances have renewed interest in mtROS as both biomarkers and therapeutic targets. Improvement in understanding of mitohormesis has refined the concept of beneficial mtROS signalling and redox-dependent adaptation [19]. Progress in nicotinamide adenine dinucleotide (NAD^+^) metabolism has highlighted a central role for NAD^+^ depletion in RGC vulnerability and RPE ageing, providing a rationale for NAD^+^-restoring interventions in ocular disease [37,38,39]. In parallel, mitochondrial-targeted compounds, including mitoquinone mesylate (MitoQ), plastoquinonyl-decyl-triphenylphosphonium (SkQ1), elamipretide (SS-31), and mitophagy and mitochondrial dynamics modulators have demonstrated neuroprotective effects in preclinical models [40,41,42]. Methodological innovations, such as redox-sensitive biosensors, quantification of mtDNA damage, Optical coherence tomography (OCT)-based metabolic indicators, and multimodal omics, now enable increasingly precise characterisation of mitochondrial dysfunction in vivo [43,44].

Nevertheless, ocular neurodegeneration arises from the interplay of genetic susceptibility, metabolic stress, vascular dysregulation, and environmental factors [45,46]. Mitochondrial dysfunction, therefore, represents a critical, but not isolated, component of a broader pathogenic network. Accumulating evidence suggests that integrative therapeutic strategies combining mitochondria-targeted interventions with neuroprotective, anti-inflammatory, and vascular-supportive approaches may more effectively address disease heterogeneity and promote sustained retinal resilience [7,15].

This review synthesises current evidence on mtROS signalling across major ocular neurodegenerative diseases and critically evaluates emerging therapeutic strategies targeting mitochondrial dysfunction. Emphasis is placed on defining the regulatory thresholds that separate adaptive from pathological mtROS signalling in retinal cells and on clarifying how progressive failure of mitochondrial quality control mechanisms drives the transition toward neurodegeneration. Key issues addressed include the molecular determinants that establish pathological mtROS thresholds across retinal cell types and the mechanistic basis for the limited clinical efficacy of broadly acting antioxidant approaches. We propose that retinal neurodegeneration is best understood as a failure of mitochondrial resilience governed by dynamic mtROS thresholds and quality control capacity, rather than a uniform oxidative stress, underscoring the importance of disease stage and mitochondrial competence for the development of precision-targeted interventions.

## 2. Disease-Specific mtROS Signatures in Ocular Neurodegeneration

mtROS constitute a central pathogenic axis across the major ocular neurodegenerative disorders, yet their roles vary according to cellular context, metabolic architecture, and disease-specific stressors. Although glaucoma, AMD, DR, and LHON share core features of mitochondrial dysfunction, including impaired oxidative phosphorylation, mtDNA instability, and redox disequilibrium, the magnitude, localisation, and temporal dynamics of mtROS production differ across retinal cell types [11,20].

RGCs are particularly vulnerable to mtROS due to their long, unmyelinated axons, which require sustained axonal transport and a high reliance on Complex I-driven oxidative phosphorylation, increasing susceptibility to oxidative injury [11,20,27]. In contrast, RPE cells are exposed to chronic photooxidative stress, intense phagocytic load, and a lipid-rich environment, which predisposes them to lipid peroxidation and complement-mediated inflammation [28]. Recognising these cell-type-specific vulnerabilities is essential for the rational design of mitochondria-targeted therapies.

Table 1 summarises the disease-specific mtROS signatures across major ocular neurodegenerative disorders, highlighting the primary sources of mtROS, vulnerable retinal cell populations, dominant mitochondrial failure mechanisms, and downstream pathological pathways [1,3,4,5,6,7,9,10,11,12,13,15,16,22,23,24,25,26,27,28,29,30,31,32,33,34,35,36,37,38,39,45,46].

### 2.1. Glaucoma

In glaucoma, progressive RGC degeneration arises from the interaction between mitochondrial vulnerability and mechanical or pressure-related stress [7,22,47,48]. Early mitochondrial dysfunction disrupts axonal transport at the lamina cribrosa, leading to local energy imbalance and increased vulnerability of distal axons to mtROS-mediated damage [49]. Impaired axonal transport limits mitochondrial replenishment, leading to the focal accumulation of complex I-derived mtROS, mtDNA damage, and the failure of mitochondrial quality control pathways [50,51,52].

Excessive Drp1-mediated mitochondrial fission combined with reduced fusion promotes mitochondrial fragmentation and bioenergetic collapse by activating apoptotic and PANoptotic pathways [53]. Age-related declines in respiratory reserve and antioxidant capacity further amplify RGC susceptibility to pressure-induced metabolic failure [25]. Together, these processes establish a self-reinforcing cycle in which mtROS act as early stress signals and accelerators of irreversible RGC degeneration.

Persistent exposure to mtROS in glaucomatous RGCs appears to promote oxidative damage to nucleic acids, particularly through the formation of 8-oxo-2′-deoxyguanosine (8-oxo-dG) lesions in both mitochondrial and nuclear DNA. mtDNA may be especially vulnerable to such injury because it lacks protective histone proteins and possesses comparatively limited repair capacity. For this reason, accumulation of 8-oxo-dG is widely considered a sensitive indicator of oxidative genomic damage in glaucoma and may reflect early disturbances in mitochondrial quality-control pathways [11,14].

Nucleotide oxidation may be particularly pronounced in RGCs owing to the distinctive metabolic demands of these neurons. RGCs rely heavily on complex I–dependent oxidative phosphorylation to maintain long-distance axonal transport. Sustained bioenergetic strain, when combined with localised ROS accumulation, could progressively compromise mtDNA integrity and thereby exacerbate mitochondrial dysfunction, reinforcing the degenerative cascade observed in glaucomatous neurodegeneration [11,27].

### 2.2. Age-Related Macular Degeneration

AMD is driven primarily by chronic mitochondrial stress within the RPE, where sustained phagocytosis of photoreceptor outer segments, cumulative photooxidative exposure, and lipofuscin accumulation generate a persistently pro-oxidative environment [11]. Mitochondrial abnormalities—including mtDNA deletions, reduced mtDNA copy number, impaired mitophagy, and defective mitochondrial biogenesis—undermine RPE metabolic capacity and promote lipid and protein oxidation [12,13,24].

Under sustained oxidative stress, lipid peroxidation products such as 4-hydroxynonenal (4-HNE) may accumulate as secondary mediators of cellular injury. This highly reactive aldehyde is generated through free-radical-mediated oxidation of polyunsaturated fatty acids, which are abundant in photoreceptor outer segments and RPE membranes. Owing to its electrophilic nature, 4-HNE readily forms covalent adducts with proteins, nucleic acids, and membrane phospholipids, processes that can alter mitochondrial enzyme activity, destabilise membrane architecture, and potentially amplify inflammatory signalling pathways [11,29].

The relative prominence of lipid-derived oxidative products in AMD likely reflects the distinctive biochemical environment of the outer retina. This region is characterised by high oxygen availability, continuous photooxidative exposure, and exceptionally lipid-rich photoreceptor membranes. Together, these factors appear to create conditions that favour membrane-centred oxidative injury.

Elevated mtROS activate complement and innate immune pathways, reinforcing chronic inflammation and contributing to drusen formation [15,23,29,30,35,54]. This progressive metabolic decline compromises photoreceptor support, establishing AMD as a paradigm of RPE-centric mtROS amplification in which mitochondrial dysfunction intersects with immune dysregulation.

### 2.3. Diabetic Retinopathy

In DR, chronic hyperglycaemia increases electron flux through the mitochondrial ETC, resulting in excess mtROS generation that overwhelms endogenous antioxidant defence [25,26]. Parallel activation of advanced glycation products (AGEs), the polyol pathway, and PKC signalling further destabilises mitochondrial redox homeostasis and bioenergetic efficiency [55,56].

In DR, mtROS-driven mitochondrial dysfunction intersects with chronic low-grade inflammation and endothelial activation, contributing to neurovascular uncoupling and progressive retinal injury. Inflammatory signalling pathways have long been recognised as central drivers of DR pathogenesis and therapeutic response [55,57]. mtROS-mediated endothelial injury disrupts tight junction integrity, induces pericyte apoptosis, and activates Müller glia, collectively impairing the blood–retinal barrier and neurovascular coupling [32,33,58]. Concurrent neuronal mitochondrial dysfunction exacerbates this vicious cycle, promoting inner retinal thinning, ischaemia, and macular oedema. Within this framework, mitochondrial-targeted interventions represent a critical adjunct to systemic glycaemic control rather than a replacement strategy [25,26,59,60].

### 2.4. Leber Hereditary Optic Neuropathy

LHON results from pathogenic mtDNA point mutations, most notably G11778A, T14484C, and G3640A, that compromise complex I activity, diminish ATP synthesis, and elevate mtROS production [6,61]. RGCs are disproportionately affected due to their high reliance on oxidative phosphorylation, long axonal structure, and limited antioxidative buffering capacity [27,28].

Impaired electron transport drives mtROS accumulation, disrupts axonal trafficking, and activates apoptotic pathways culminating in rapid RGC loss [62]. Environmental factors such as smoking further intensify mtROS-mediated injury, highlighting the interplay between genetic predisposition and modifiable risks [6]. Current therapies, including idebenone, aim to partially restore electron flow and reduce mtROS. However, variable clinical efficacy underscores the need for strategies that more robustly enhance mitochondrial resilience [63,64].

### 2.5. Cross-Disease Integration of mtROS Mechanisms

**Figure 1:**
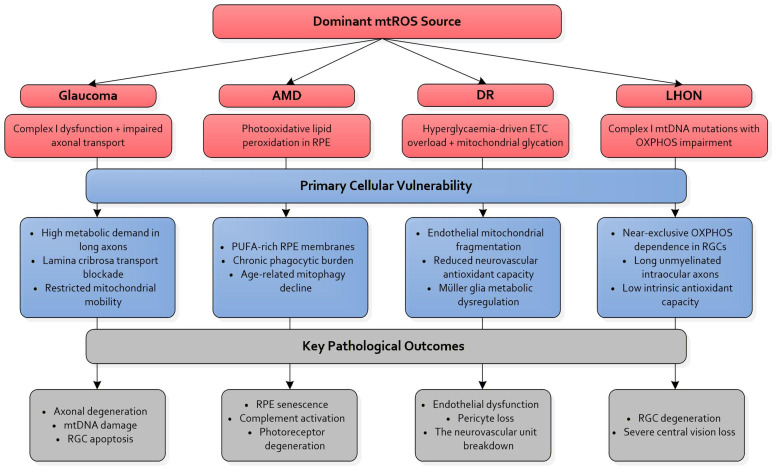
Disease-specific mtROS sources and signalling pathways in ocular neurodegeneration. The figure highlights the spatial and temporal distribution of mtROS across major ocular diseases, integrating complex I/III-derived ROS, lipid peroxidation burden, mitophagy failure, and neurovascular stress. It contrasts RGC-predominant vulnerability in glaucoma and LHON with RPE-centric oxidative injury in AMD and neurovascular disruption in DR. Together, these patterns illustrate how distinct pathological triggers converge on a common axis of mtROS amplification and mitochondrial quality control failure. mtROS: Mitochondrial reactive oxygen species; AMD: Age-related macular degeneration; DR: Diabetic retinopathy; LHON: Leber’s hereditary optic neuropathy; RPE: Retinal pigment epithelium; ETC: electron transport chain; OXPHOS: oxidative phosphorylation; RGCs: Retinal ganglion cells; PUFA: Polyunsaturated fatty acids.

Figure 1 shows a comparative schematic of mtROS signatures across the major ocular neurodegenerative diseases—glaucoma, AMD, DR, and LHON—highlighting both shared and disease-specific sites of mitochondrial vulnerability [1,3,4,5,6,7,9,10,15,16,23,24,25,26,27,28,29,30,31,32,33,34,35,36,37,38,39,45,46,63,64].

Across these disorders, a convergent theme emerges: mtROS become pathogenic when mitochondrial quality control capacity is exceeded, generating disease-specific yet mechanistically unified patterns of degeneration [11,27]. RGCs are highly sensitive to disruptions in complex I activity and axonal transport, whereas RPE cells undergo mtROS-mediated lipid peroxidation and complement activation under chronic photooxidative pressure [13,29]. In DR, hyperglycaemia-driven mitochondrial overload destabilises endothelial and neuronal redox homeostasis [32,57], while LHON represents a genetically primed state in which complex I defects push mtROS beyond compensatory thresholds [28].

This unified framework underscores mtROS as an upstream driver of ocular neurodegeneration and supports therapeutic strategies designed to restore mitochondrial resilience rather than merely suppress ROS. Together, these observations support the concept that mtROS-driven retinal neurodegeneration reflects a failure of adaptive buffering capacity rather than uniform oxidative stress, reinforcing the need for precision, stage-dependent mitochondrial interventions.

Although individual retinal disorders exhibit distinct biomarker profiles, several oxidative indicators appear to represent shared manifestations of mitochondrial stress that become differentially expressed depending on cellular and metabolic context. Oxidative nucleotide lesions such as 8-oxo-dG may arise in multiple retinal pathologies because mtDNA is inherently susceptible to reactive oxygen species-mediated damage [11,14]. Their relative abundance, however, appears to vary according to the dominant metabolic pressures in specific retinal cell populations.

In glaucoma and inherited optic neuropathies, RGCs rely heavily on complex I–dependent oxidative phosphorylation, a metabolic configuration that may render mtDNA particularly vulnerable to oxidative modification [11,27]. By contrast, lipid peroxidation products such as 4-HNE are often more evident in AMD. This observation likely reflects the lipid-rich architecture of photoreceptor outer segments and the chronic photooxidative conditions characteristic of the RPE [11,29].

These observations suggest that oxidative biomarkers should not necessarily be interpreted as disease-specific entities but rather as context-dependent manifestations of a broader redox stress network [14,27]. Recognising this convergence-divergence pattern could improve biomarker-based patient stratification and may ultimately support the development of metabolically informed therapeutic strategies.

## 3. Mitochondrial ROS Biology in the Retina: Signalling, Sources, and Thresholds

Mitochondria are central regulators of energy metabolism and redox homeostasis in retinal cells, which rank among the most metabolically active tissues in the human body. Sustained oxidative phosphorylation, high oxygen consumption, and continuous exposure to light induce oxidative stress, making retinal neurons and supporting cells especially vulnerable to mtROS generation [11,18,20]. The retina’s unique combination of high O_2_ demand, abundant polyunsaturated fatty acids (PUFAs), and exposure to photo-oxidative stress underscores its sensitivity to redox imbalance and makes it an ideal model for studying the paradoxical roles of oxygen in physiology and pathology [21].

Importantly, mtROS are not merely toxic by-products of respiration but function as essential signalling molecules that regulate stress adaptation, survival pathways, and mitochondrial quality control. Dysregulation of mtROS production or detoxification, however, contributes directly to retinal pathologies, including AMD, DR, glaucoma, and retinitis pigmentosa [11,18,20].

### 3.1. Cellular and Molecular Sources of mtROS in Retinal Cells

The mitochondrial ETC represents the dominant source of mtROS, with complexes I and III being the principal contributors. Complex I generate substantial ROS during RET, a process that occurs when the ubiquinone pool is highly reduced, and mitochondrial membrane potential is elevated. Under these conditions, electrons flow backwards toward complex I, promoting the formation of superoxide. While tightly regulated reverse electron transport (RET) contributes to physiological redox signalling, dysregulated RET is a potent driver of oxidative stress in retinal cells [34].

At complex III, superoxide generated at the Qo site can be released into both the mitochondrial matrix and the intermembrane space, facilitating the diffusion of ROS toward the cytosol when antioxidant buffering capacity is exceeded [11]. Chronic ETC-derived ROS overproduction induces lipid peroxidation, protein oxidation, and mtDNA damage, thereby accelerating retinal degeneration [65]. In contrast, moderate ROS levels modulate apoptosis, autophagy, and pro-survival signalling pathways, underscoring the context-dependent nature of ETC-derived mtROS.

### 3.2. Physiological Roles of mtROS in Retinal Homeostasis

Beyond ETC-associated electron leakage, several enzymatic and lipid-associated processes contribute to mtROS generation. Mitochondrial NADPH oxidase (NOX) isoforms generate superoxide through NADPH-dependent oxygen reduction, while monoamine oxidase (MAO) localised on the outer mitochondrial membrane produces hydrogen peroxide during neurotransmitter metabolism [11,18].

The retina is enriched in polyunsaturated fatty acids, rendering photoreceptor and RPE cells particularly vulnerable to lipid peroxidation and oxidative damage [21]. Lipid-derived ROS propagate oxidative damage through self-amplifying chain reactions, contributing to photoreceptor dysfunction and AMD pathogenesis [66]. Conversely, mitochondrial uncoupling proteins (UCPs) mitigate excessive ROS production by partially dissipating the proton gradient, thereby limiting electron leakage and supporting retinal cell survival [67]. Collectively, these convergent mtROS sources highlight the intrinsic retinal vulnerability to redox imbalance and the necessity for precise regulation of mitochondrial redox signalling.

### 3.3. Experimental Tools for mtROS Detection: Methodological Constraints and Translational Implications

Recent advances in genetically encoded biosensors have significantly enhanced our ability to monitor mtROS dynamics in retinal cells in real-time [65]. Redox-sensitive green fluorescent protein (roGFP) enables ratiometric quantification of the intracellular redox state, whereas the roGFP Orp1 and HyPer variants provide selective, real-time monitoring of hydrogen peroxide dynamics. MitoSOX remains widely used for detecting mitochondrial superoxide, although its irreversible oxidation, phototoxicity, and susceptibility to artefacts necessitate cautious interpretation. MitoTimer provides complementary information on cumulative mitochondrial protein oxidation and turnover [68,69].

Despite these advances, significant methodological limitations persist. Distinguishing RET-derived ROS from ROS generated during forward electron transport at complexes I or III in vivo remains technically challenging [34,70]. Pharmacological inhibitors such as rotenone and antimycin A disrupt ETC function broadly and may induce compensatory ROS production, complicating mechanistic attribution [68,71]. In addition, confocal and multiphoton imaging of retinal tissue is constrained by limited optical access, rapid ROS kinetics, and challenges in quantitative calibration [72,73].

Importantly, these methodological constraints have direct translational implications. While current biosensors provide valuable mechanistic insight, their integration into clinically actionable biomarker strategies remains limited. Bridging this gap will require alignment of mtROS imaging approaches with non-invasive clinical readouts, such as OCT-based metabolic indicators, retinal oximetry, and aqueous humour biomarkers, to enable patient stratification and early detection of mitochondrial stress [74].

### 3.4. Threshold-Dependent mtROS Signalling and Pathological Consequences

mtROS play a dual role in retinal cells, acting as essential signalling molecules under physiological conditions while contributing to oxidative damage when produced in excess [10,21,67,75]. In the retinal environment, tightly regulated mtROS signalling is required to maintain mitochondrial function, cellular homeostasis, and neuronal survival [9,76].

Importantly, mtROS signalling does not follow a linear dose–response relationship but is governed by threshold-dependent dynamics. Low to moderate mtROS levels support redox-sensitive signalling pathways and adaptive stress responses, whereas sustained or excessive mtROS generation promotes mitochondrial dysfunction, oxidative damage, and neurodegenerative cascades [75,77,78]. The transition between these states is determined by the efficiency of antioxidant systems and mitochondrial quality control mechanisms [19,79].

Within this framework, mitohormesis represents the physiological manifestation of threshold-dependent mtROS signalling, describing adaptive cellular responses elicited below pathogenic transition points, where mitochondrial quality control pathways remain functional [80,81]. These adaptive responses enhance cellular resilience to metabolic and oxidative stress without triggering irreversible damage.

When mtROS levels exceed cellular buffering capacity, however, adaptive signalling progressively shifts toward maladaptive amplification. Excess mtROS impair mitochondrial dynamics, compromise mitophagy, and promote the accumulation of dysfunctional mitochondria, thereby reinforcing oxidative stress and accelerating retinal neurodegeneration [22,52,82,83]. This pathological transition underscores the central role of mtROS thresholds in determining disease progression.

Collectively, threshold-dependent mtROS signalling provides a unifying mechanistic link between physiological redox communication and mitochondrial quality control failure in retinal neurodegenerative diseases, forming the conceptual basis for stage-specific and mitochondria-targeted therapeutic strategies [84].

### 3.5. Convergent mtROS-Driven Mechanisms Across Retinal Diseases

When mtROS production overwhelms antioxidant defences, convergent pathological cascades are initiated across retinal diseases. mtDNA, located in proximity to the ETC, is highly susceptible to oxidative damage. Accumulated mtDNA mutations impair respiratory efficiency and perpetuate ROS generation. Oxidised mtDNA fragments released into the cytosol act as danger-associated molecular patterns (DAMPs), activating innate immune pathways such as the NLRP3 inflammasome and amplifying retinal inflammation [85,86,87].

Oxidised lipids and proteins compromise membrane integrity, enzymatic activity, and intracellular signalling, further propagating inflammatory and degenerative responses [16,35]. In parallel, ROS-induced disruption of mitochondrial fission–fusion dynamics promotes accumulation of dysfunctional organelles, reinforcing oxidative stress and bioenergetic failure [88]. Chronic mtROS-driven inflammation and mitochondrial network collapse thus represent shared pathogenic mechanisms across AMD, glaucoma, and DR [16,35]. Collectively, these observations position mitochondria as both guardians and executioners of retinal health, with cellular fate determined by the balance between mtROS generation, detoxification capacity, and adaptive signalling thresholds.

### 3.6. ER–Mitochondria Communication and Lipid Redox Signalling in Retinal Stress

The endoplasmic reticulum (ER) and mitochondria form a closely coordinated signalling system that helps regulate cellular adaptation to metabolic and proteostatic stress. Their functional interaction occurs primarily at mitochondria-associated ER membranes (MAMs), specialised contact regions that facilitate controlled calcium exchange and redox communication between the two organelles. Through these interfaces, mitochondrial metabolism becomes tightly linked to ER protein-folding capacity and intracellular calcium homeostasis. In retinal cells, this relationship appears particularly significant, as mitochondrial bioenergetics is highly sensitive to fluctuations in ER-derived calcium flux [89].

ER stress activates the unfolded protein response (UPR). This adaptive signalling program restores proteostasis but may initiate apoptotic pathways if cellular homeostasis cannot be re-established [90]. Emerging evidence suggests that disturbances in ER calcium handling may precede overt mitochondrial dysfunction in several retinal disease contexts. Such upstream perturbations could influence both the magnitude and timing of mtROS production, implying that mitochondrial oxidative stress should be interpreted within a broader ER–metabolic stress axis rather than as an isolated pathogenic event.

Local regulatory systems may further modulate this interface. Endogenous factors such as melatonin signalling and steroidogenic pathways appear capable of influencing mitochondrial redox balance and ER–mitochondria coupling efficiency [91]. Through these mechanisms, local modulators could partially buffer ER stress by sustaining mitochondrial function and limiting excessive mtROS generation [92]. Nevertheless, this compensatory capacity is likely finite. When ER stress becomes prolonged or severe, adaptive signalling may gradually collapse, allowing pro-apoptotic pathways to dominate and thereby reinforcing mitochondrial dysfunction.

From a biomarker perspective, these observations suggest a potential temporal hierarchy in which ER-derived stress signals emerge before measurable mitochondrial depolarisation or substantial increases in mtROS. Several studies indicate that disturbances in ER proteostasis and metabolic balance often precede detectable mitochondrial impairment [93,94,95]. Integrating ER stress markers with mitochondrial and metabolic readouts may therefore enhance early disease detection and improve patient stratification within precision-medicine frameworks [94]. Although mitochondrial parameters such as mtROS remain widely used indicators of disease progression, they frequently become detectable only after upstream metabolic disturbances have already developed [93,96].

Within this broader redox context, lipid peroxidation products such as 4-HNE and F2-isoprostanes provide complementary insight into oxidative damage affecting membrane structures. These molecules arise predominantly from the non-enzymatic oxidation of polyunsaturated fatty acids within membrane phospholipids and therefore tend to reflect membrane-level oxidative injury rather than exclusively primary mitochondrial dysfunction. F2-isoprostanes are produced through free-radical-mediated peroxidation of arachidonic acid and are widely considered reliable indicators of in vivo lipid oxidative damage [97].

By contrast, prostaglandins such as PGF2α are generated enzymatically through cyclooxygenase activity and participate in regulated physiological processes, including inflammation and vascular tone. Clinically, PGF2α analogues such as latanoprost are widely used in glaucoma therapy to reduce intraocular pressure by enhancing aqueous humour outflow. Distinguishing non-enzymatic lipid oxidation from regulated prostaglandin signalling is therefore important for accurate biomarker interpretation and therapeutic design [98].

## 4. Mitochondrial Quality Control Failure in Ocular Neurodegeneration

Mitochondrial integrity in retinal cells is maintained by an interconnected network of quality control mechanisms that regulate redox balance, organelle turnover, and proteostasis. In RGCs, photoreceptors, and RPE cells, sustained mitochondrial stress arising from continuous oxidative phosphorylation, lipid-rich membranes, and light-induced oxidative insults necessitates efficient quality control function for cellular survival [20,82,83,99].

These systems encompass enzymatic antioxidant defences, redox-responsive transcription governed by nuclear factor erythroid 2-related factor 2 (Nrf2) [21,75,83], dynamic regulation of mitochondrial fusion, fission, and mitophagy [48], and proteostatic mechanisms such as the mitochondrial UPR (UPRmt). Mounting evidence indicates that disruption of these tightly integrated pathways constitutes a central driver of mitochondrial dysfunction and neurodegeneration in glaucoma, AMD, DR, and inherited optic neuropathies [22,48,82].

### 4.1. Antioxidant Enzymes and Redox Buffering Systems in the Retina

ROS are unavoidable by-products of oxidative phosphorylation, generated predominantly at complexes I and III of the ETC. To limit oxidative damage while preserving physiological redox signalling, retinal cells rely on a multilayered and highly coordinated antioxidant network [100].

Superoxide dismutase 2 (SOD2), localised within the mitochondrial matrix, constitutes the first line of defence by catalysing the conversion of superoxide into hydrogen peroxide (H_2_O_2_) [101]. Reduced SOD2 activity leads to excessive mtROS accumulation, accelerating photoreceptor and RPE degeneration and contributing to AMD-like pathology and inherited mitochondrial disorders [13,102].

Detoxification of H_2_O_2_ and lipid peroxides is mediated primarily by glutathione peroxidases (GPX1 and GPX4), which utilise reduced glutathione (GSH) as an electron donor [103]. Glutathione reductase regenerates GSH from its oxidised form using NADPH, sustaining redox buffering capacity [104]. Because the neural retina is enriched in polyunsaturated fatty acids and subjected to intense photo-oxidative stress, continuous GSH recycling is essential. GSH depletion sensitises photoreceptors to oxidative injury and promotes AMD-like pathology [105,106], whereas enhanced GPX4 expression confers strong protection against retinal degeneration and ferroptotic stress [12,107].

Catalase, although predominantly peroxisomal, can translocate to mitochondria under oxidative stress and catalyses the decomposition of H_2_O_2_ into water and oxygen [108,109]. Age-associated declines in catalase activity increase RPE vulnerability and contribute to the pro-oxidative environment characteristic of AMD [110].

Complementing the glutathione system, the mitochondrial thioredoxin 2 (Trx2) pathway provides rapid redox buffering. Trx2, maintained in its reduced state by TrxR2, donates electrons to peroxiredoxin 3 (Prx3), enabling efficient detoxification of H_2_O_2_. In DR, chronic hyperglycaemia disrupts NADPH availability, impairing Trx2–Prx3 activity and sustaining mtROS accumulation [59,111]. Collectively, these enzymatic systems form a dynamic redox-buffering network that preserves mitochondrial integrity while permitting adaptive redox signalling in retinal cells.

### 4.2. Nrf2 Signalling and Redox-Responsive Transcription

Nrf2 functions as the master regulator of antioxidant and cytoprotective gene expression. Under basal conditions, Nrf2 is sequestered by Kelch-like ECH-associated protein 1 (Keap1) and targeted for proteasomal degradation. Oxidative stress modifies reactive Keap1 cysteine residues, stabilising Nrf2 and permitting its nuclear translocation, where it activates transcription driven by the antioxidant response element (ARE). Nrf2 target genes include SOD2, GPx isoforms, Prx3/5, enzymes involved in glutathione biosynthesis, and NADPH-generating dehydrogenases, which are essential for maintaining cellular redox homeostasis [112,113]. In the retina, Nrf2 confers protection to both RPE cells and photoreceptors against photo-oxidative stress and lipid peroxidation. Genetic ablation of Nrf2 accelerates RPE degeneration, promotes drusen-like deposits, induces chronic inflammation, and disrupts mitochondrial metabolism, recapitulating key features of human AMD [114,115].

Beyond antioxidant defence, Nrf2 directly interfaces with mitochondrial quality control pathways. Stress-induced Nrf2 activation upregulates p62, an autophagy adaptor that facilitates mitophagic clearance of damaged mitochondria [116]. Nrf2 also enhances the expression of mitochondrial chaperones and proteases, which support proteostasis and mitochondrial recovery [78,82]. Through these mechanisms, Nrf2 integrates redox control with mitochondrial turnover and metabolic adaptation, positioning it as a central integrator of retinal mitochondrial resilience.

### 4.3. Mitochondrial Dynamics and Mitophagy in Retinal Neuroprotection

Mitochondrial dynamics maintain organelle function through continuous cycles of fusion and fission, facilitating redistribution of mitochondrial components and isolation of damaged regions. Fusion is mediated by mitofusin 1 and 2 (MFN1/2) at the outer membrane and optic atrophy 1 (OPA1) at the inner membrane, promoting respiratory efficiency, mtDNA complementation, and dilution of oxidative stress [117,118]. Disruption of MFN2 or OPA1 results in mitochondrial fragmentation, impaired oxidative phosphorylation, and heightened stress vulnerability, with direct relevance to glaucoma and LHON [119]. Fission, driven primarily by dynamin-related protein 1 (DRP1), enables mitochondrial distribution and quality control [120]. However, excessive DRP1 activation promotes pathological fragmentation and energetic failure, as observed in DR and ischemic retinal injury [121,122].

Mitophagy selectively removes dysfunctional mitochondria and is indispensable for retinal homeostasis [79]. In the PTEN-induced kinase 1 (PINK1)/Parkin pathway, mitochondrial depolarisation stabilises PINK1 on the outer membrane, triggering Parkin-mediated ubiquitination and autophagic clearance [123]. Reduced PINK1/Parkin activity leads to accumulation of defective mitochondria in RPE cells and contributes to the progression of AMD [124].

Receptor-mediated mitophagy via BCL2 Interacting Protein 3 (BNIP3) and NIX is particularly relevant under hypoxic or inflammatory conditions [125]. To maintain mitochondrial population size, mitophagy must be counterbalanced by mitochondrial biogenesis. Peroxisome proliferator-activated receptor-gamma coactivator 1-alpha (PGC-1α) orchestrates this process by promoting mtDNA replication and synthesis of respiratory proteins [126]. Reduced PGC-1α expression in AMD and glaucoma compromises mitochondrial renewal, which accelerates neurodegeneration [20].

### 4.4. The Mitochondrial Unfolded Protein Response

UPRmt is activated by the accumulation of misfolded or unimported proteins within the mitochondrial matrix or inner membrane [99,127]. UPRmt induces nuclear transcription of mitochondrial chaperones, proteases, antioxidant enzymes, and metabolic regulators who restore proteostasis and support organelle recovery [128].

Transcription factors: activating transcription factor (ATF5), ATF4, and C/EBP homologous protein (CHOP) integrate UPRmt with the integrated stress response and Nrf2 signalling, linking proteostatic stress to broader cellular adaptation pathways [129,130].

In retinal cells, transient UPRmt activation enhances resistance to photo-oxidative and metabolic stress. However, sustained mitochondrial dysfunction can shift UPRmt signalling toward pro-apoptotic programmes, contributing to RPE degeneration and progression of AMD and DR. Thus, UPRmt represents a context-dependent quality control mechanism that balances adaptive recovery against elimination of irreversibly damaged cells. Mitochondrial quality control in the retina relies on tightly coordinated antioxidant defences, mitophagy, and proteostatic mechanisms [82,131]. The core molecular components that constrain mtROS signalling in retinal cells are summarised in Table 2, highlighting the key quality control processes that preserve redox balance at the level of individual mitochondria [12,16,17,19,28,81,82,99,112,122,124,125].

## 5. Molecular Modulators of Mitochondrial ROS and Quality Control

Therapeutic strategies aimed at modulating mitochondrial redox homeostasis encompass both pharmacological and non-pharmacological approaches that enhance mitochondrial resilience through complementary mechanisms. Growing evidence suggests that integrative therapeutic strategies combining mitochondria-targeted interventions with neuroprotective, anti-inflammatory, and vascular-supportive approaches may more effectively address disease heterogeneity and promote sustained retinal resilience. In this context, environmental enrichment has emerged as a promising non-pharmacological strategy for enhancing mitochondrial function, reducing oxidative stress, and supporting neuroprotection in retinal models, providing a complementary avenue to conventional drug-based therapies [8].

The biological impact of mtROS is determined not by absolute abundance but by a dynamic equilibrium between redox signalling, mitohormetic adaptation, and antioxidant defences coordinated by Sirtuin 1 (SIRT1), Nrf2, and PGC-1α [80,122]. Retinal neurons rely on an extensive network of endogenous antioxidant systems and mitochondrial quality-control pathways to buffer physiological mtROS signalling and preserve mitochondrial integrity under conditions of sustained metabolic stress [11,16,18,20,42]. Table 3 summarises the principal mitochondrial quality control modules that collectively determine the transition from adaptive mtROS signalling to maladaptive amplification [12,15,19,35,36,81,82,86,112,122,125].

### 5.1. Natural Compounds and Nutraceutical Modulators

Natural antioxidants and nutraceuticals exert pleiotropic effects on mitochondrial function, redox balance, and inflammatory signalling pathways relevant to retinal neurodegeneration. Compounds such as resveratrol, curcumin, sulforaphane, nicotinamide, and idebenone primarily converge on SIRT1-, Nrf2-, and PGC-1α-dependent pathways, which collectively regulate oxidative stress responses, mitochondrial biogenesis, and metabolic flexibility in ocular tissues [132,133,134,135].

Resveratrol is among the most extensively investigated nutraceutical modulators of mitochondrial function. SIRT1 activation promotes mitochondrial biogenesis, suppresses excessive ROS production, and enhances metabolic adaptability [136,137,138]. In experimental models of AMD and DR, resveratrol reduces oxidative burden and improves RGC survival [136]. Anti-inflammatory effects mediated via NF-κB inhibition have been demonstrated in uveitis [139] while ocular surface studies indicate improved mitochondrial efficiency [140]. However, poor bioavailability and limited ocular penetration remain the primary translational barriers, necessitating advanced delivery strategies such as nanoparticle-based or conjugated formulations [135,141].

Curcumin robustly activates Nrf2-ARE signalling by disrupting Keap1–Nrf2 interactions [142,143]. Upregulation of heme oxygenase-1 (HO-1) and NAD(P)H quinone dehydrogenase 1 (NQO1) enhances retinal resistance to oxidative stress. It confers protection in preclinical models of AMD and DR, attenuating inflammation and mitigating photoreceptor stress [133,144,145,146]. As resveratrol, curcumin is limited by low systemic stability and poor solubility, reinforcing the need for advanced ocular delivery approaches.

Sulforaphane (SFN), a potent isothiocyanate-derived Nrf2 inducer, enhances detoxification pathways and promotes autophagic clearance via transcription factor EB (TFEB) activation [147,148]. In experimental models of AMD and DR, SFN delays RPE and photoreceptor degeneration [149]. However, uncertainties regarding optimal dosing, solubility, and ocular pharmacokinetics currently limit clinical applicability [44].

Nicotinamide replenishes intracellular NAD^+^ pools, which are essential for mitochondrial metabolism, DNA repair, and SIRT1-mediated signalling [39,150]. Preclinical studies further confirm robust structural and functional protection of RGCs [151]. However, long-term dosing strategies, interindividual metabolic variability, and safety thresholds remain incompletely defined [51].

Idebenone, a short-chain benzoquinone and synthetic analogue of coenzyme Q10, enhances electron transport efficiency and attenuates ROS production under conditions of impaired mitochondrial respiration. Its clinical relevance is best established in LHON, where it bypasses dysfunctional complex I and reduces excessive superoxide generation [63,64]. Idebenone also modulates RET-associated ROS bursts, a key contributor to optic nerve vulnerability in mitochondrial optic neuropathies. Partial success in LHON clinical trials underscores mechanistic precision, while highlighting interindividual variability and the need for predictive biomarkers [64,152].

Collectively, nutraceuticals exert biologically meaningful and mechanistically diverse effects on retinal mitochondria but face substantial translational challenges related to bioavailability, pharmacokinetics, and insufficient retinal targeting. Their future clinical utility likely resides in combination strategies incorporating mitochondria-targeted therapeutics or NAD^+^-restorative interventions.

### 5.2. Mitochondria-Targeted Antioxidants and Peptides

Mitochondria-targeted antioxidants, including MitoQ, SkQ1, and the mitochondria-protective peptide SS-31, are designed to neutralise oxidative injury directly at the site of mtROS generation. MitoQ and SkQ1 exploit triphenylphosphonium (TPP^+^)-driven electrophoretic accumulation within mitochondria, a process dependent on the inner mitochondrial membrane potential [153,154]. Through this targeting strategy, MitoQ reduces ROS production and improves mitochondrial bioenergetics in experimental models of retinal degeneration. Efficacy may nevertheless be attenuated in advanced stages of retinal neurodegeneration, where progressive mitochondrial dysfunction limits therapeutic responsiveness. Furthermore, dose-dependent systemic toxicity has been reported for MitoQ, constraining clinical escalation and necessitating cautious optimisation of treatment regimens [40,154,155].

SkQ1 demonstrates potent antioxidant and mitochondria-protective effects in preclinical models of retinal degeneration [154,155,156,157]. Nevertheless, differences between systemic and topical delivery efficacy underscore the ongoing difficulty of attaining adequate retinal penetration without advanced formulation strategies. In contrast, SS-31 employs a membrane-potential-independent mechanism by selectively binding cardiolipin within the inner mitochondrial membrane. This interaction stabilises ETC supercomplexes, improves mitochondrial efficiency, and reduces apoptotic signalling [158]. Robust neuroprotection has been demonstrated in glaucoma models by reducing RGC apoptosis [159,160].

Clinically, SS-31 represents the most advanced mitochondria-targeted therapy in ophthalmology. In the Phase II RECLAIM-2 trial (NCT03891875) involving patients with dry AMD, SS-31 subcutaneous treatment resulted in statistically significant improvements in visual function and deceleration of photoreceptor degeneration, as evidenced by a reduced rate of ellipsoid zone loss, while maintaining an acceptable safety and tolerability profile [161]. Ongoing Phase III trials (ReNEW and ReGAIN) will further define long-term efficacy. Nevertheless, the need for intravitreal administration remains a significant obstacle to widespread adoption, highlighting the importance of developing less invasive yet equally efficient delivery platforms.

Collectively, mitochondria-targeted antioxidants and peptides provide a strong mechanistic rationale and robust preclinical efficacy but remain limited by delivery-related constraints. Overcoming these barriers will require advanced ocular delivery strategies, including nanoparticle-based systems, sustained-release formulations, and non-invasive mitochondrial targeting approaches.

### 5.3. NAD^+^ Modulators and Bioenergetic Regulation

NAD^+^-enhancing compounds, including nicotinamide, nicotinamide mononucleotide, and nicotinamide riboside, represent a promising class of metabolic therapies for retinal neuroprotection. These agents act as precursors of NAD^+^, a central coenzyme governing cellular metabolism, mitochondrial function, and neuronal survival. Their pharmacokinetic profiles and mechanisms of action suggest potential applications in ocular neurodegenerative conditions such as glaucoma and AMD [37,38,162,163].

Restoration of NAD^+^ supports oxidative phosphorylation, DNA repair, and mitochondrial dynamics—processes central to RGC survival. Nicotinamide remains the most clinically validated precursor, rapidly increasing NAD^+^ levels and improving mitochondrial transport, morphology, and efficiency in RGCs, with confirmed functional recovery in glaucoma patients [37,162].

Nicotinamide mononucleotide reduces cellular senescence and mitochondrial damage in RPE cells, mechanisms relevant to early AMD, while nicotinamide riboside confers similar metabolic benefits, albeit with more limited ocular-specific data [38,163].

Emerging NAD^+^ analogues and tetrahydroquinoxaline derivatives offer enhanced stability, bioavailability, and tissue selectivity. However, unresolved questions regarding long-term safety, tissue-specific NAD^+^ responses, and the theoretical risk of supporting pathological proliferation remain to be addressed [164].

### 5.4. Emerging Experimental Mitochondrial Interventions

Experimental approaches targeting mitochondrial dysfunction include mitochondria-penetrating peptides (MPPs), gene-based strategies such as allotropic expression, pharmacological enhancement of mitophagy, and modulation of nitric oxide (NO) signalling [9,42,52,165,166,167]. MPPs facilitate direct delivery of therapeutic cargo into mitochondria, improving intracellular bioavailability while limiting systemic exposure. Their precision targeting demonstrates encouraging preclinical potential for retinal disorders, where enhanced mitochondrial uptake may overcome existing delivery barriers [165].

Gene-based strategies, particularly allotropic expression, relocate mitochondrial gene transcription to the nucleus, enabling nuclear synthesis and subsequent mitochondrial import of functional proteins. This approach provides a mechanism to bypass pathogenic mtDNA mutations and is particularly relevant for mitochondrial optic neuropathies, including LHON [166].

Mitophagy enhancers promote selective removal of dysfunctional mitochondria, thereby reducing oxidative stress and preserving neuronal viability in glaucoma and DR models. However, excessive activation may destabilise cellular metabolism, underscoring the need for precise therapeutic control [52,167].

Aberrant NO signalling further contributes to mitochondrial impairment in ocular tissues. Consequently, NOX inhibitors targeting dysregulated NO pathways are being explored for their potential to reduce oxidative injury and support mitochondrial function in models of retinal and optic nerve disease [167].

Despite encouraging preclinical findings, efficient tissue-specific delivery, minimisation of off-target effects, and demonstration of long-term safety remain essential prerequisites for clinical translation of these experimental modalities.

### 5.5. Mechanistic Constraints and Opportunities: Hormesis, RET, and Redox Biosensing

Adaptive, threshold-dependent mtROS responses define the non-linear relationship between ROS levels and cellular resilience. Subthreshold ROS elevation activates Nrf2-, SIRT1-, and mitochondrial biogenesis pathways, enhancing retinal resistance to injury. In contrast, excessive ROS levels trigger apoptosis and neurodegeneration [13,80,81]. Understanding these hormetic response curves is critical for defining therapeutic windows, as indiscriminate antioxidant intervention may inadvertently suppress beneficial redox signalling [122,168].

**Figure 2:**
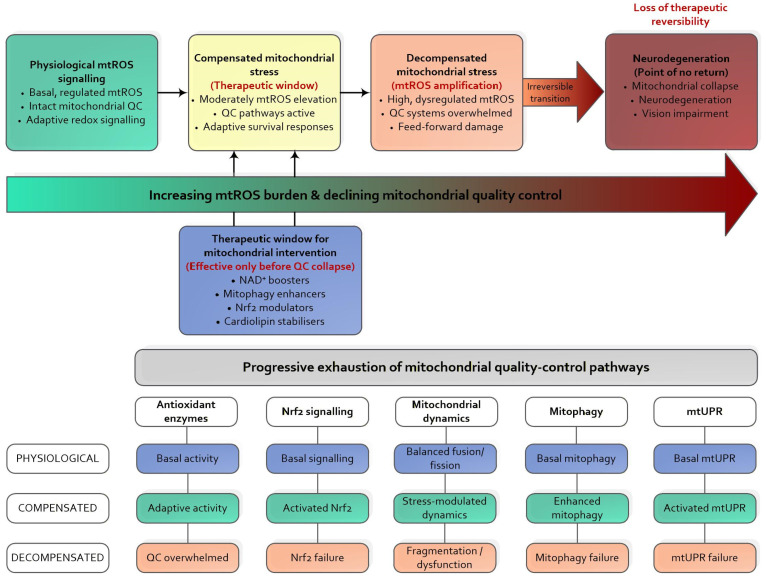
Threshold-dependent mtROS signalling in retinal neurodegeneration. Physiological mtROS maintain retinal homeostasis under intact mitochondrial quality control. During compensated mitochondrial stress, moderately elevated mtROS trigger adaptive responses, defining a therapeutic window for precise mtROS modulation. Exhaustion of antioxidant defences, mitochondrial dynamics, and mitophagy transforms mtROS into self-amplifying drivers of dysfunction, leading to irreversible neuronal loss beyond the threshold. mtROS: Mitochondrial Reactive Oxygen Species; QC: Mitochondrial Quality Control; NAD: Nicotinamide adenine dinucleotide; Nrf2: Nuclear factor erythroid 2–related factor 2; PGC-1α: Peroxisome proliferator-activated receptor gamma coactivator 1-alpha; MitoQ: Mitoquinone mesylate; SkQ1: plastoquinonyl-decyl-triphenylphosphonium; DRP1: Dynamin-related protein 1; MFN1/2: Mitofusin 1/2; OPA1: Optic Atrophy 1; BNIP3/BNIP3L: BCL2/adenovirus E1B 19 kDa interacting protein and BNIP3-like protein; UPRmt: Mitochondrial unfolded protein response.

To conceptualise this non-linearity during retinal disease progression, Figure 2 illustrates a continuum from physiological mtROS signalling to retinal neurodegeneration driven by progressive failure of mitochondrial quality control mechanisms. This framework integrates threshold-dependent mtROS signalling, RET-dependent mtROS amplification, and the progressive narrowing of the therapeutic window for effective redox-targeted interventions [15,17,18,19,20,36,37,38,39,40,41,42,50,75,122].

NAD^+^ availability plays a central role in mitochondrial metabolism and redox balance. As a key coenzyme in energy production, NAD^+^ supports mitochondrial function and regulates mitophagy through its influence on SIRT1 activity, which governs mitochondrial biogenesis and stress responses. Reduced NAD^+^ levels impair these regulatory mechanisms, compromising mitochondrial quality and increasing disease susceptibility [169].

Closely linked to this process is mitophagic efficiency. Mitophagy enables the selective removal of damaged mitochondria, thereby preventing the accumulation of dysfunctional organelles and limiting oxidative stress. The PINK1–PRKN pathway represents one of the well-characterised regulatory mechanisms of mitophagy, and its disruption has been strongly associated with neurodegenerative disorders [15,169].

Another crucial determinant is the balance between ROS and antioxidant defences. Mitochondrial metabolism continuously produces ROS, which, if insufficiently neutralised, can damage mtDNA, proteins, and membranes. Adequate antioxidant reserves, therefore, play a protective role in maintaining mitochondrial integrity and preventing oxidative injury [170,171].

Mitochondrial membrane potential is essential for ATP synthesis and ionic homeostasis. Its disruption represents a hallmark of mitochondrial dysfunction and can trigger the release of pro-apoptotic factors, ultimately initiating cell death pathways [172].

These mechanisms are highly interconnected. Alterations in one regulatory component often propagate through others, collectively determining the threshold at which mitochondrial dysfunction becomes pathological. Consequently, effective therapeutic strategies may require integrated approaches targeting multiple dimensions of mitochondrial quality control simultaneously.

RET-generated ROS represent a physiologically regulated yet potentially pathological burst of superoxide formed during reverse electron flow at complex I [63,64,152].

Advances in mitochondrial biosensors, including mtROS probes, NAD^+^/NADH ratio sensors, and mitophagy reporters, are refining the mechanistic insight of these pathways. However, in vivo application remains constrained by phototoxicity, probe instability, and challenges in discriminating mitochondrial from cytosolic ROS, underscoring the need for standardised metabolic readouts and improved biosensor design [52,65,82,148,173].

## 6. Strategies for Mitochondria-Targeted Therapeutic Intervention in the Eye

The high degree of cellular specialisation and compartmentalisation within retinal neurons further complicates subcellular targeting strategies. Although recent advances in local administration techniques, nanocarrier engineering, and sustained-release systems have increased the feasibility of mitochondrial delivery, substantial biological and technological barriers continue to limit clinical implementation [36,174,175].

### 6.1. Barriers to Ocular and Mitochondrial Targeting

Drug delivery to the posterior segment of the eye is intrinsically constrained by multiple anatomical and physiological barriers, including the cornea, conjunctival epithelium, blood–aqueous barrier, and blood–retinal barrier [176]. Topical administration is particularly limited by rapid tear film turnover, nasolacrimal drainage, and restricted transcorneal permeability, resulting in negligible retinal drug concentrations [177]. Systemic delivery is similarly hindered by the tight junctions of the blood–retinal barrier, rendering local administration strategies essential for effective therapy in the posterior segment [174,175].

Mitochondrial targeting introduces an additional layer of complexity. The inner mitochondrial membrane maintains a highly negative membrane potential (ΔΨm), which restricts entry of hydrophilic or anionic molecules while favouring electrophoretic accumulation of lipophilic cations such as TPP^+^ [153,178]. This principle underlies many mitochondria-targeted antioxidants. In dysfunctional mitochondria, ΔΨm decreases, thereby reducing membrane potential-dependent uptake; this limitation contributes to neurodegeneration, especially the loss of RGCs in glaucoma and the oxidative injury that characterises AMD [42,179]. This inconsistency highlights the need for delivery systems that can function under conditions of mitochondrial stress and depolarisation.

### 6.2. Local Routes of Ocular Administration

Topical eye drops offer a non-invasive and patient-friendly route of administration but deliver negligible quantities of drugs to the posterior segment, confining their utility largely to anterior segment disorders [180,181]. Intravitreal injection remains the clinical gold standard for retinal drug delivery, providing direct access to the vitreous cavity and enabling diffusion toward RGCs and photoreceptors. While mitochondria-targeted compounds such as MitoQ and SkQ1 are conceptually suitable for intravitreal delivery, long-term in vivo evidence demonstrating sustained mitochondrial accumulation remains limited. Moreover, repeated intravitreal injections increase the risk of complications, including endophthalmitis, retinal detachment, and cumulative patient burden [182].

Subretinal administration enables highly efficient delivery to photoreceptors and the RPE and is therefore preferred for gene therapies targeting outer retinal degeneration. However, its surgical invasiveness and spatially restricted transduction limit widespread retinal coverage [183]. Suprachoroidal delivery has recently emerged as a minimally invasive alternative for distributing therapeutics across the choroid and outer retina. Preliminary studies suggest potential applicability for mitochondrial modulators requiring broad retinal penetration, although direct evidence supporting mitochondrial targeting via this route remains limited [184].

### 6.3. Carrier and Delivery Technologies

Advances in nanoengineering have enabled the development of diverse carrier systems designed to enhance mitochondrial targeting in ocular tissues. Liposomes, composed of biocompatible phospholipid bilayers, can encapsulate both hydrophilic and lipophilic agents and may be functionalised with TPP^+^ or mitochondria-targeting peptides to promote mitochondrial accumulation [87,185,186]. Polymeric nanoparticles, including poly(lactic-co-glycolic acid) (PLGA) systems, provide tunable size, biodegradability, and sustained drug release. These features are particularly advantageous for chronic retinal diseases requiring prolonged mitochondrial modulation [187,188].

Lipophilic cations, particularly TPP^+^ derivatives, function as mitochondrial address tags that exploit the negative ΔΨm to enhance accumulation of conjugated antioxidants, thereby improving protection against oxidative retinal injury [189,190]. MPPs offer a complementary targeting strategy that does not rely on membrane potential, enabling translocation across mitochondrial membranes even under depolarising conditions. This property renders MPPs promising carriers for proteins, nucleic acids, and small molecules in diseases characterised by mitochondrial dysfunction [25,165].

### 6.4. Sustained-Release and Implantable Systems

Given the chronic and progressive nature of mitochondrial dysfunction in ocular neurodegenerative diseases, sustained exposure to mitochondria-targeted therapeutics is essential for durable neuroprotection. Biodegradable intravitreal implants, microspheres, and polymer-based delivery systems enable maintenance of therapeutic drug levels over extended periods while reducing the frequency of invasive interventions [135]. Existing clinical implants, such as dexamethasone-releasing devices used in macular oedema and AMD, provide a translational precedent for adapting similar platforms to deliver mitotropic agents to RGCs and photoreceptors [191,192].

Stimuli-responsive materials, including hydrogels and redox-sensitive polymers, are designed to release therapeutic cargo in response to oxidative stress, pH shifts, or other microenvironmental cues associated with mitochondrial injury. Such adaptive release strategies may enhance both efficacy and safety by concentrating drug exposure during periods of heightened mitochondrial vulnerability [193,194,195].

### 6.5. Unresolved Challenges in Retina-Specific Mitochondrial Targeting

Despite substantial technological progress, multiple challenges continue to limit effective mitochondrial targeting in the retina [153,178]. Achieving selective targeting of RGCs, photoreceptors, or RPE cells remains difficult, as each cell type exhibits distinct mitochondrial architectures and metabolic environments that influence carrier uptake and intracellular trafficking [154,178,186].

Following cellular internalisation, carriers must escape endosomal compartments and traverse the cytosol to reach mitochondria, a process that remains inefficient for many nanoparticles and peptide-based systems [25,165]. In addition, oxidative conditions within diseased retinal tissue may destabilise carriers or degrade sensitive therapeutics, further reducing effective mitochondrial delivery [26,42,73].

Excessive accumulation of therapeutic agents in mitochondria also raises safety concerns, as disruption of physiological ROS signalling, and ETC function may occur [122,179]. Chronic modulation of mitochondrial pathways lacks comprehensive toxicological and pharmacodynamic evaluation, particularly regarding effects on mtDNA integrity, mitophagy, and long-term metabolic adaptation [12,13,36].

Collectively, these unresolved challenges emphasise the need for delivery platforms capable of operating under mitochondrial stress, maintaining stability in oxidative environments, and achieving precise subcellular targeting without compromising mitochondrial homeostasis. Continued refinement of these technologies is essential for enabling clinically meaningful mitochondrial-targeted interventions in ocular neurodegenerative disease. As these limitations shape the development of next-generation mitochondrial therapeutics, careful evaluation of experimental models, functional endpoints, and emerging biomarkers will be critical for guiding translational progress.

## 7. Experimental Models, Functional Endpoints, and Translational Biomarkers

Given the complexity of mitochondrial dysfunction and redox regulation in retinal neurons and supporting cells, no single model can fully capture disease-relevant mechanisms across spatial and temporal scales. Therefore, a combination of in vitro, in vivo, and clinically translatable functional endpoints is required to bridge mechanistic insight with therapeutic development and regulatory approval.

### 7.1. In Vitro Models of Retinal Mitochondrial Dysfunction

In vitro retinal models constitute a fundamental platform for probing mitochondrial mechanisms, confirming target engagement, delineating safety profiles, and evaluating candidate therapeutics prior to in vivo validation. Conventional two-dimensional (2D) cultures have been widely used since the early 20th century due to their reproducibility, low cost, and suitability for long-term and high-throughput studies [196]. However, 2D systems fail to reproduce the three-dimensional architecture, cellular heterogeneity, and extracellular matrix interactions characteristic of retinal tissue, limiting their ability to model complex mitochondrial stress responses and intercellular metabolic coupling.

Primary RPE cells isolated from adult or fetal human eye cups provide a closer resemblance to native RPE physiology, including polarisation and barrier function [197]. Nevertheless, their limited proliferative capacity and progressive acquisition of mesenchymal features restrict long-term applicability. Immortalised RPE lines, including adult retinal pigment epithelial cell line (ARPE-19) and human telomerase reverse transcriptase-immortalised retinal pigment epithelial-1 (TERT-RPE1), were developed to overcome these constraints. However, ARPE-19 cells exhibit variable differentiation capacity, altered gene expression profiles, and phenotype drift across culture conditions and passages, reducing reproducibility across laboratories [198].

More recently, RPE differentiation from embryonic stem cells (ESCs) has emerged as a robust alternative. ESC-derived RPE cells demonstrate high morphological, functional, and transcriptional similarity to native RPE and are particularly suitable for drug screening and transepithelial transport assays [199].

Beyond monolayer cultures, human stem cell–derived retinal organoids (ROs) have transformed in vitro retinal disease modelling. First reported in 2011, optic vesicle-like structures derived from ESCs and induced pluripotent stem cells (iPSCs) self-organise into laminated retinal tissue containing all major neuronal cell types and Müller glia, recapitulating retinal development, morphogenesis, and polarity [200]. Integration with microfluidic platforms further enables modelling of biochemical gradients and metabolic stress, positioning ROs as next-generation tools for studying mitochondrial dysfunction and screening mitochondria-targeted therapies.

### 7.2. In Vivo Models of Retinal Neurodegeneration

In vivo models remain indispensable for evaluating disease progression, systemic responses, and therapeutic safety. DR models include chemically induced diabetes via streptozotocin (STZ) or alloxan, dietary interventions, laser-induced injury, and genetically modified strains [201]. STZ-induced diabetes is most commonly used due to rapid disease onset, whereas dietary models require longer durations. Genetically modified mice such as Ins2Akita, db/db, and non-obese diabetic strains model specific metabolic components of DR. Combined models, exemplified by the Akimba mouse (VEGF overexpression crossed with Akita diabetes), better recapitulate features of both early and advanced DR. Although most models predominantly reflect non-proliferative stages [201].

Animal models of AMD have been developed in rodents, rabbits, pigs, and non-human primates. Rodent models offer low cost and genetic tractability but lack a macula, a major anatomical limitation [202]. Nevertheless, key AMD-associated features—including Bruch’s membrane thickening, subretinal deposits, chronic inflammation, and microglial activation—have been reproduced through genetic manipulation of complement pathways (CFH, C3), chemokines (Ccl2, Cxcr1), and antioxidant defences (Sod1, Sod2), as well as through dietary and laser-based approaches [202].

Glaucoma models exploit both genetic predisposition and induced ocular hypertension. The DBA/2J mouse develops a spontaneous age-related increase in intraocular pressure, progressive RGC loss, and optic nerve degeneration. Additional transgenic models express mutations in Myoc, optineurin, or Cyp1b1, facilitating dissection of specific pathogenic mechanisms [203]. No single in vivo model captures the full spectrum of glaucoma pathology; however, models that combine mitochondrial stress with mechanical or metabolic insults are most informative for studying early mtROS dysregulation.

### 7.3. Functional and Molecular Endpoints

Robust functional and molecular endpoints are essential for assessing mitochondrial integrity and therapeutic efficacy. Mitophagy plays a central role in maintaining mtDNA quality, particularly in heteroplasmic disorders where mutant mtDNA burden determines disease severity. Quantification of mitophagy remains technically challenging, requiring integration of multiple approaches [204].

Clinical studies underscore the translational relevance of retinal mitochondrial endpoints. In patients with genetically defined mitochondrial diseases, reduced visual acuity and full-field electroretinography (fERG) amplitudes correlate with decreased outer nuclear layer volume on spectral-domain optical coherence tomography (SD-OCT), connecting structural degeneration to mitochondrial dysfunction [205].

Imaging biomarkers further enhance mechanistic insight, underscoring the need for biomarker-guided identification of therapeutic windows. Hyperreflective foci (HRF) on OCT represent a validated risk marker for AMD progression and correspond to hyperautofluorescent signals on fundus autofluorescence (FAF) imaging. Fluorescence lifetime imaging ophthalmoscopy (FLIO) enables spectral and temporal characterisation of FAF, revealing prolonged lifetimes and hypsochromic emission shifts during RPE dysmorphia and migration, suggesting early metabolic alterations preceding structural degeneration [206,207].

Molecular analyses demonstrate that mtDNA damage accumulates with normal ageing but is markedly exacerbated in AMD. Karunadharma et al. demonstrated region-wide mtDNA lesion accumulation in AMD RPE exceeding nuclear DNA damage by nearly eightfold, supporting mitochondrial dysfunction as a core pathogenic driver [208]. Mitophagy assessment employs Western blotting, immunohistochemistry, reporter mice (e.g., LC3-based fluorescent models), and transmission electron microscopy, which remains the gold standard despite high cost and limited tissue availability [204,208].

### 7.4. Biomarkers for Clinical Translation

Translational biomarkers connecting mitochondrial function to clinical outcomes are critical for therapeutic development. Flavoprotein fluorescence (FPF) imaging enables non-invasive assessment of mitochondrial redox state, with elevated intensity and heterogeneity observed in diabetic eyes and proliferative DR, correlating with visual impairment [209]. Unlike purely structural imaging, FPF provides metabolically targeted insight into retinal mitochondrial dysfunction.

MicroRNAs (miRNAs) have emerged as additional biomarkers and therapeutic targets. Dysregulated miRNA profiles influence mitochondrial dynamics, oxidative stress responses, and cell survival pathways across retinal diseases, offering mutation-independent indicators of disease progression and therapeutic response [210].

For instance, miR-96 and miR-182 contribute to retinal degeneration by affecting mitochondrial function in photoreceptor cells. Dysregulated miRNAs in retinal diseases, therefore, show potential as biomarkers of mitochondrial dysfunction in ocular pathologies [210].

Despite their promise, clinical application remains challenging. Variability in miRNA expression across disease stages and conditions complicates their reliability as biomarkers. Further research is required to standardise miRNA-based diagnostics and therapeutics across diseases [211,212].

## 8. Clinical and Regulatory Perspectives on Retinal Mitochondrial Dysfunction

Mitochondria-targeted interventions for ocular neurodegenerative diseases are increasingly advancing from preclinical validation toward clinical evaluation. This transition introduces distinct regulatory, methodological, and translational challenges that arise from mitochondria’s pleiotropic roles in cellular metabolism, redox signalling, calcium homeostasis, inflammation, and cell survival [14,41]. Therapeutic modulation of mitochondrial function, therefore, requires an integrated clinical framework that aligns mechanistic rationale, patient selection, endpoint definition, delivery strategies, and long-term safety monitoring.

### 8.1. Clinical Trials Targeting Mitochondrial Dysfunction in Ocular Diseases

Clinical translation of mitochondrial therapies has progressed most prominently in inherited optic neuropathies, particularly LHON, in which primary pathogenic mechanisms directly involve mtDNA mutations impairing oxidative phosphorylation. Gene therapy strategies designed to restore respiratory chain function have yielded the most compelling clinical evidence to date. Five-year follow-up data from lenadogene nolparvovec gene therapy demonstrated sustained visual improvement or stabilisation in a subset of patients with ND4-associated LHON, supporting the durability of mitochondrial rescue at the level of RGCs [213].

Additional clinical trials continue to explore gene replacement strategies targeting mitochondrial complex I subunits, including ND1-associated LHON (Neurophth Therapeutics Inc., NCT05820152) and the Gene Therapy Clinical Trial for the Treatment Of Leber’s HereDitary Optic Neuropathy (GOLD) trial conducted by Wuhan Neurophth Biotechnology (NCT04912843). Collectively, these studies establish LHON as a leading proof-of-concept indication for mitochondrial therapeutics, supported by its monogenic aetiology, relative preservation of retinal architecture at early disease stages, and availability of quantifiable functional visual endpoints [63,213].

Beyond gene therapy, pharmacological modulation of mitochondrial structure and bioenergetics has entered clinical evaluation. Elamipretide (SS-31), a mitochondria-targeted tetrapeptide that stabilises cardiolipin and enhances ETC efficiency, has been investigated in randomised clinical trials in LHON. While primary visual acuity endpoints have shown variable responses, secondary outcome measures indicate potential benefits on retinal function, mitochondrial bioenergetic stability, and stress resilience [209]. These observations emphasise the importance of disease stage, therapeutic timing, and the selection of sensitive functional and metabolic endpoints when assessing mitochondrial interventions in neurodegenerative conditions.

Broader analyses of LHON clinical trials further highlight the need for harmonised outcome measures, refined patient stratification based on mutation type and residual mitochondrial reserve, and extended follow-up durations to capture delayed or cumulative mitochondrial recovery [118]. Together, these clinical experiences provide a translational template for extending mitochondrial therapeutic strategies to more complex, multifactorial retinal diseases.

### 8.2. Pharmacological Complexity and Nutraceutical Approaches

Mitochondria-targeted compounds (MTCs) exert multifaceted biological effects due to the highly interconnected nature of mitochondrial processes, including redox balance, ATP synthesis, calcium handling, and apoptotic signalling. Consequently, both therapeutic efficacy and safety profiles are strongly dose-dependent. Mild uncoupling of oxidative phosphorylation may attenuate mtROS generation and confer cytoprotective effects. However, excessive uncoupling or high-dose antioxidant exposure can impair ETC function, disrupt physiological redox signalling, or induce pro-oxidant effects [178,214].

To date, the majority of preclinical and clinical data in ocular disease have been generated using lipophilic triphenylphosphonium-based antioxidants, such as MitoQ and SkQ1, or cardiolipin-stabilising peptides such as elamipretide. While these agents validate mtROS as a therapeutically relevant target, their limited clinical penetration and variable efficacy highlight the need for next-generation mitochondria-targeted molecules with improved specificity, pharmacokinetics, tissue distribution, and long-term safety profiles [40,189].

In parallel, nutraceuticals have gained increasing attention as adjunctive strategies for mitochondrial support in ocular neurodegeneration. Nutraceuticals may serve as adjunctive strategies for mitochondrial support in chronic retinal disease [138,146]. Despite generally favourable safety profiles, clinical translation remains constrained by poor bioavailability, variable ocular tissue penetration, and inconsistent dosing regimens. These limitations are largely governed by physicochemical properties, including solubility, lipophilicity, and molecular weight, which critically influence intestinal absorption and cellular uptake [215].

Nevertheless, nutraceuticals represent a potentially valuable complementary approach, particularly for chronic retinal diseases requiring long-term intervention. Clinical implementation will require rigorously designed, adequately powered trials incorporating standardised formulations, controlled dosing strategies, and mitochondrial-specific functional endpoints [214,215].

### 8.3. Roadmap for Clinical Translation

Future clinical translation of mitochondrial therapies in ophthalmology is expected to benefit from a phased, indication-specific development strategy. Early-phase clinical trials are most likely to succeed in diseases characterised by a strong mitochondrial aetiology, preserved retinal structure, and availability of sensitive functional and metabolic biomarkers, as exemplified by inherited optic neuropathies [41,64]. Patient stratification based on genetic background, metabolic reserve, and disease stage is anticipated to enhance therapeutic signal detection and reduce clinical heterogeneity.

Combination therapeutic strategies represent a promising direction. In multifactorial disorders such as AMD and DR, mitochondria-targeted interventions may complement existing standards of care, including anti-VEGF therapies, by addressing neurodegenerative, metabolic, and inflammatory components that remain insufficiently targeted by existing treatments [14,50].

From a regulatory perspective, long-term safety monitoring is essential given the central role of mitochondria in systemic cellular homeostasis. Clinical trial designs should incorporate assessment of systemic exposure, off-target effects, and potential interference with physiological redox signalling. The integration of advanced retinal imaging modalities, functional mitochondrial biomarkers, and refined ocular delivery systems is expected to facilitate regulatory evaluation and accelerate approval pathways for mitochondria-directed therapies [175,187,207].

Collectively, these considerations position mitochondrial therapeutics as a transformative yet technically and regulatory demanding frontier in ophthalmology, with successful translation dependent on precision targeting, rational combination strategies, and biomarker-informed patient selection.

## 9. Key Knowledge Gaps and Research Priorities

mtROS constitute a central regulatory axis in retinal homeostasis, integrating cellular metabolism, stress adaptation, and neuroinflammatory signalling [63]. While major advances have clarified mitochondrial contributions to glaucoma, AMD, DR, and LHON, critical conceptual and translational gaps continue to limit therapeutic progress [9,22,42,48,148,216]. Addressing these gaps requires a shift from descriptive pathology toward quantitative, cell-type–resolved, and clinically actionable models of mitochondrial dysfunction [138,217].

### 9.1. The Central Paradox of mtROS Signalling

A persistent conceptual challenge arises from the dual and context-dependent nature of mtROS. Under physiological conditions, mtROS function as signalling intermediates that regulate redox-sensitive transcription, mitochondrial biogenesis, ion homeostasis, and adaptive mitophagy, thereby sustaining retinal neurons in physiological conditions while preserving redox adaptability [17,19,20]. When mitochondrial quality control systems remain intact, these signals promote metabolic flexibility and cellular resilience.

In contrast, mitochondrial ETC impairment, lipid peroxidation, and mtDNA instability shift mtROS signalling toward maladaptive amplification, triggering inflammasome activation, ferroptosis, and apoptotic cascades [12,15,16,35]. Importantly, this transition is neither linear nor uniform across retinal cell types or disease contexts. Defining vulnerability thresholds that delineate adaptive from pathological mtROS signalling remains a critical unmet need. Establishing this “therapeutic window”, a redox range in which interventions enhance resilience without disrupting essential signalling, is therefore a priority for translational progress [19,81,122].

Achieving this goal will require integrative experimental platforms that can capture the rapid, subcellular-scale fluctuations of mtROS in vivo. Ratiometric biosensors, single-cell redox imaging, multiphoton metabolic microscopy, and combined OCT/ERG functional readouts remain underutilised despite their capacity to define actionable boundaries between adaptive and pathological mtROS signalling [34,65,72].

### 9.2. Mitochondrial Heterogeneity Across the Retina

The retina comprises a mosaic of mitochondrial phenotypes shaped by distinct structural and functional requirements across retinal cell types. RGCs rely heavily on axonal transport and NAD^+^ metabolism, while photoreceptors operate at near-maximal energetic throughput. The RPE exhibits a unique susceptibility to lipid oxidation and defective mitophagy [13,18,28,29]. Despite advances in single-cell and spatial transcriptomics, mitochondrial proteomics, and metabolomics, the field lacks a unified atlas integrating metabolic, spatial, and redox parameters across health and disease states [43,44].

Such an atlas must resolve mitochondrial states across neuronal, glial, and vascular compartments, while capturing disease stage-specific transitions and microenvironmental influences. Current inconsistencies in sample preparation, sequencing pipelines, and computational analyses limit the comparability of findings across studies. Standardisation of mitochondrial profiling, integration of high-resolution metabolic imaging, and application of predictive computational models are therefore essential steps toward precision mitochondrial medicine in ophthalmology [20,74,82].

### 9.3. Therapeutic Opportunities and Limitations in Retinal mtROS Dysfunction

Although mitotropic antioxidants, NAD^+^ boosters, modulators of mitochondrial dynamics, mitophagy regulators, and gene-based interventions show promise in preclinical models, their clinical translation remains limited. Barriers include insufficient intravitreal penetration, variable uptake into RGC and RPE mitochondria, and heterogeneous mitochondrial membrane potentials that unpredictably influence drug accumulation and efficacy [153,178,186].

To contextualise therapeutic strategies within the trajectory of progressive mitochondrial dysfunction, Table 4 summarises stage-specific interventions alongside their principal limitations [1,7,15,17,18,19,20,22,23,24,28,34,35,36,40,41,42,45,46,50,75,122,180]. This framework emphasises that therapeutic efficacy is tightly constrained by disease stage and mitochondrial quality control capacity, and that mistimed interventions may attenuate benefit or exacerbate dysfunction.

The table conceptualises retinal neurodegeneration as a stage-dependent continuum defined by mitochondrial quality control capacity and mtROS signalling state. Therapeutic efficacy is critically dependent on disease stage, with the greatest potential for benefit residing within a narrow therapeutic window characterised by partially preserved mitochondrial QC. Interventions applied outside their appropriate stage may attenuate benefit or exacerbate dysfunction, underscoring the importance of precise patient stratification and timing.

Emerging delivery platforms, including nanoparticles, lipophilic conjugates, suprachoroidal formulations, and mitochondria-targeting vectors such as triphenylphosphonium and cardiolipin-binding peptides, offer new opportunities to overcome these barriers [165,180,194]. However, their mitochondrial pharmacokinetics, long-term retention, and off-target redox effects remain poorly characterised. Establishing standardised protocols for mitochondrial drug profiling is therefore a prerequisite for rational therapeutic development [12,122,179].

### 9.4. Biomarkers for Patient Stratification and Early Detection

Effective translation of mtROS-targeted therapies requires biomarkers capable of detecting mitochondrial stress before irreversible neurodegeneration occurs. Conventional structural and electrophysiological metrics primarily capture late-stage damage and lack sensitivity to early metabolic decline [32,73]. Candidate biomarkers include aqueous humour mtDNA parameters, heteroplasmy burden, mitochondrial-derived peptides, oxidative damage signatures, metabolic ratios, retinal oximetry, and OCT-A-based microvascular metrics linked to mitochondrial dysfunction [12,13,44].

Recent precision-medicine approaches in DR further emphasise the importance of genetic and epigenetic biomarkers in defining disease trajectories and therapeutic responsiveness, reinforcing the need for mitochondrial- and redox-informed patient stratification frameworks [54]. Integrating these measures into composite mitochondrial stress indices may enable biomarker-enriched clinical trials and improve patient stratification [43].

### 9.5. Collaborative Frameworks for Translational Research

Traditional clinical trial designs often lack mitochondrial biomarkers, metabolic endotyping, and early functional endpoints, limiting their capacity to detect meaningful neuroprotection. Adaptive, biomarker-guided trial frameworks anchored in mitochondrial dysfunction rather than broad phenotypic classifications may provide a more efficient translational pathway [41]. Progress will depend on interdisciplinary collaboration spanning molecular biology, ophthalmology, pharmacology, computational science, and regulatory medicine, supported by harmonised methodologies and validated mitochondrial endpoints [13,28].

## 10. Future Directions for Precision Mitochondrial Therapies

Advancing therapeutic strategies for retinal neurodegeneration will likely require a transition toward precision mitochondrial medicine, in which interventions are adapted to the disease stage and to the specific mitochondrial susceptibilities of individual patients. A growing body of evidence suggests that mitochondrial dysfunction may represent a common pathogenic axis across several major ocular neurodegenerative disorders, including glaucoma, AMD, DR, and inherited optic neuropathies [8,9,164]. Nevertheless, the mechanisms underlying mitochondrial impairment appear to differ across diseases and across stages of progression, underscoring the importance of biomarker-guided therapeutic strategies.

Progress in retinal imaging technologies, together with advances in spatial and single-cell omics, and the identification of mitochondrial biomarkers, provides promising opportunities to detect early signatures of mitochondrial stress and to characterise patient-specific risk profiles [42,75,122]. Such approaches may allow mitochondrial dysfunction to be recognised before irreversible neuronal loss occurs, thereby potentially widening the therapeutic window for intervention.

Future therapeutic strategies will likely involve stage-specific and combinatorial approaches. Mitochondria-targeted antioxidants, NAD^+^ modulators, and agents that regulate mitochondrial dynamics or mitophagy have demonstrated encouraging effects in experimental models, suggesting that targeted modulation of mitochondrial quality-control pathways may help preserve neuronal viability and delay functional deterioration [50,75,122].

A clearer operational definition of therapeutic windows for mitochondrial-targeted interventions may emerge through the integration of candidate biomarkers, advanced imaging metrics, and metabolic profiling. Collectively, these approaches may help delineate the balance between the beneficial and potentially detrimental consequences of therapies that influence mitochondrial quality-control processes, particularly mitophagy.

Candidate biomarkers represent an initial layer of clinical evaluation. For example, mtROS levels may provide insight into mitochondrial integrity and oxidative stress, thereby enabling assessment of the physiological consequences of therapies designed to modulate mitophagy [218]. In parallel, proteins directly involved in mitophagy, such as PINK1 and Parkin, could serve as molecular indicators of mitochondrial turnover and quality-control activity [219].

Imaging strategies further complement biomarker analyses by enabling the visualisation of mitochondrial activity within living tissues. Advanced modalities, including positron emission tomography (PET), may enable the assessment of mitochondrial integrity and metabolic function, thereby providing valuable information regarding therapeutic responses and tissue-specific metabolic adaptations [220].

Metabolic profiling offers an additional analytical perspective. Metabolomic evaluation of mitochondria-associated metabolites may reveal alterations in cellular energy metabolism and oxidative stress, helping to identify metabolic shifts associated with therapeutic interventions [221].

Despite the potential therapeutic benefits of stimulating mitophagy, excessive mitochondrial turnover could disrupt cellular metabolic homeostasis. This concern may be particularly relevant in tissues with high energetic demands, such as the heart, retina, and brain, emphasising the need to define therapeutic windows that are both safe and biologically effective [219,222].

Translational progress will also require addressing substantial clinical challenges, including the development of efficient drug-delivery strategies targeting retinal tissues and the establishment of reliable approaches for monitoring mitochondrial responses in vivo.

Emerging findings additionally suggest that therapeutic strategies may benefit from integrating mitochondria-directed pharmacological interventions with broader systemic approaches that support cellular resilience. Interventions that promote mitochondrial biogenesis, metabolic flexibility, and redox homeostasis could potentially complement conventional treatments and contribute to more sustained neuroprotection. In this context, environmental enrichment has been proposed as a systems-level strategy capable of enhancing mitochondrial performance, reducing oxidative stress, and activating neuroprotective pathways in retinal models, thereby representing a potential non-pharmacological adjunct to mitochondria-targeted therapies [8].

Taken together, these developments imply that future progress in this field may rely on integrated, biomarker-informed therapeutic frameworks that align mitochondrial interventions with disease stage, cellular susceptibility, and systemic metabolic context.

## 11. Conclusions

Ocular neurodegenerative diseases are increasingly recognised as a major clinical and societal challenge. A substantial body of research suggests that mitochondrial dysfunction represents a shared pathogenic feature across several retinal disorders.

Rather than arising solely from uniform oxidative injury, retinal degeneration appears to involve a progressive disruption of mitochondrial quality-control systems. These systems maintain redox equilibrium, regulate organelle turnover, and support metabolic adaptation.

Within this framework, mtROS species appear to fulfil a dual biological role. At physiological levels, mtROS participate in redox signalling and cellular adaptation. When produced in excess or inadequately regulated, however, mtROS may contribute to mitochondrial instability and increased neuronal vulnerability. Recognition of this threshold-dependent behaviour of mtROS is therefore central to the rational design of therapeutic interventions.

Accordingly, effective therapeutic strategies may need to focus not on indiscriminate suppression of reactive oxygen species but on the precise recalibration of mitochondrial redox signalling and restoration of mitochondrial quality-control capacity.

Durable neuroprotection will likely depend on identifying mitochondrial vulnerability thresholds and implementing biomarker-guided therapeutic strategies. Early intervention before mitochondrial homeostasis deteriorates beyond the point of recovery may be particularly important.

Shifting attention from generalised oxidative stress toward mitochondrial resilience and adaptive redox regulation may provide a useful conceptual framework for developing more effective therapeutic approaches to retinal neurodegeneration. Future research integrating mitochondrial biology, precision diagnostics, and targeted therapeutic modulation may ultimately enable earlier intervention and more durable neuroprotection in retinal neurodegenerative diseases.

## Figures and Tables

**Table 1:** Disease-specific mitochondrial ROS and failure mechanisms in retinal neurodegeneration.

Disease	Primary Vulnerable Cell Type(s)	Dominant mtROS Sources	Key Mitochondrial Failure Mechanisms	Characteristic Downstream Pathways	Representative Biomarkers	References
Glaucoma	RGCs Lamina cribrosa cells	Complex I dysfunction RET Axonal transport blockade Excitotoxic ROS	Enhanced fission (Drp1) Reduced fusion (Mfn1/2) mtDNA oxidation NAD^+^ depletion Impaired mitophagy (PINK1/Parkin)	Axonal degeneration PANoptosis NLRP3 inflammasome activation Chronic neuroinflammation	8-oxo-dG Reduced Complex I activity Decreased NAD^+^ Increased MitoSOX signal	[1,7,9,10,15,16,27,28,34,35,36,37,38]
AMD	RPE Secondary photoreceptors	Photooxidative stress Lipid peroxidation Complex I/III leakage A2E oxidation	mtDNA deletions Impaired PGC-1α biogenesis Defective β-oxidation Cardiolipin oxidation	Complement activation (C3/C5) Drusen formation Lipid–protein adducts Metabolic uncoupling	4-HNE MDA Complement fragments mtDNA deletions Oxidised A2E	[3,23,24,29,30,31,37,39,43]
DR	Endothelial cells Pericytes Müller glia	ETC overload PKC–AGE–RAGE signalling polyol pathway flux NOX2/NOX4 activation	Mitochondrial swelling/depolarisation Increased fission (Drp1) Reduced fusion (OPA1) Impaired mitophagy Glutathione depletion	BRB breakdown Pericyte loss Neurovascular uncoupling Microaneurysm formation	AGEs Elevated MitoSOX ROS Reduced Complex IV activity Decreased GSH	[4,12,25,26,32,33,45,46]
LHON	RGCs	Pathogenic mtDNA mutations (Complex I) Excessive superoxide RET-driven amplification	Severe ATP deficiency Respiratory supercomplex destabilisation Secondary mtDNA damage	Rapid RGC degeneration Optic atrophy Heightened stress sensitivity	Pathogenic mtDNA variants Reduced Complex I activity F2-isoprostanes Increased superoxide	[5,6,27,28,34,35,36]

RGCs: Retinal ganglion cells; mtROS: mitochondrial reactive oxygen species; Drp1: Dynamin-related protein 1; 8-oxo-Dg: 8-okso-2′-deoksigvanozin; RET: reverse electron transport; Mfn 1/2: mitofusin 1 and 2; mtDNA: mitochondrial DNA; NAD: nicotinamide adenine dinucleotide; NLRP3: NOD-like receptor family pyrin domain containing 3; PINK1: PTEN-induced kinase 1; MitoSOX: mitochondrial superoxide; RPE: retinal pigment epithelium; PGC-1α: peroxisome proliferator-activated receptor-gamma coactivator 1-alpha; 4-HNE: 4-Hidroksinonenal; MDA: malondialdehyde; A2E: ETC: electron transport chain; PKC-AGE-RAGE: Advanced Glycation End-products (AGEs) bind to their Receptor (RAGE), activating intracellular signaling involving Protein Kinase C (PKC); NOX: Mitochondrial NADPH oxidase; OPA1: optic atrophy 1; BRB: Blood–retinal barrier; ROS: reactive oxygen species; ATP: Adenosine triphosphate; GSH: Glutathione.

**Table 2:** Core mitochondrial quality control components regulating mtROS in retinal cells.

Quality Control Process	Key Components	Primary Function	Impact on mtROS Regulation	References
Antioxidant defence	SOD2, PRDX3/5, GPX1/4	Detoxifies superoxide and peroxides	Limits excessive mtROS while maintaining physiological signalling	[12,19,122]
Redox-responsive transcription	Nrf2, PGC-1α, Nrf1/2	Induces antioxidant enzymes and mitochondrial biogenesis	Adjusts mtROS buffering to metabolic/oxidative demand	[17,81,112]
Mitochondrial dynamics	MFN1/2, OPA1, DRP1	Regulates fusion–fission balance	Constrains or amplifies mtROS via mitochondrial domain segregation	[28,82,122]
Mitophagy initiation	PINK1, Parkin, BNIP3, BNIP3L/NIX	Removes damaged mitochondria	Prevents propagation of high mtROS mitochondria	[99,124,125]
Proteostasis and stress response	LONP1, CLPP, UPRmt	Removes misfolded mitochondrial proteins	Maintains ETC integrity Limits mtROS leakage	[19,112,124]
Lipid redox control	GPX4, cardiolipin remodelling enzymes	Suppresses mitochondrial lipid peroxidation	Prevents lipid-driven mtROS escalation and ferroptotic/inflammatory signalling	[12,16,19]

SOD2: superoxide dismutase 2; PRDX3/5: peroxidoxin 3 and 5; GPX1/4: glutathione peroxidases 1 and 4; mtROS: mitochondrial reactive oxygen species; Nrf2: nuclear factor erythroid 2-related factor 2; PGC-1α: peroxisome proliferator-activated receptor-gamma coactivator 1-alpha; MFN1/2: mitofusin 1 and 2; OPA1: optic atrophy 1; DRP1: dynamin-related protein 1; PINK1: PTEN induced kinase 1; BNIP3: BCL2 Interacting Protein 3; BNIP3L: BCL2/adenovirus E1B 19 kDa-interacting protein 3-like; BCL2/adenovirus E1B 19 kDa-interacting protein 3-like; BCL2: Interacting Protein 3 Like; LONP1: lon protease 1; CLPP: caseinolytic protease P; UPRmt: mitochondrial unfolded protein response; ETC: Electron transport chain.

**Table 3:** Molecular modulators of mitochondrial ROS and quality control in retinal cells.

QC Network	Integrated Processes	Functional Outcome	Failure Consequence	References
Redox–metabolic coupling	Antioxidant systems + metabolic sensing	Adaptive mtROS signalling Metabolic flexibility	Energetic imbalance Maladaptive mtROS amplification	[19,122]
Organelle turnover network	Mitochondrial dynamics + mitophagy	Renewal of mitochondrial population	Accumulation of dysfunctional mitochondria	[82,122,125]
Stress-adaptive transcription	Nrf2, PGC-1α, UPRmt signalling	Expansion of mitochondrial capacity under stress	Exhaustion of adaptive responses	[17,81,112]
Lipid and membrane integrity	Cardiolipin homeostasis + GPX4 activity	Protection against lipid-driven mtROS escalation	Ferroptosis Inflammatory signalling	[12,16,19]
Mitochondria–inflammation axis	mtROS, mtDNA release Inflammasome activation	Controlled immune signalling	Chronic neuroinflammation Neuronal loss	[15,35,36,86]

QC: quality control; mtROS: mitochondrial reactive oxygen species; Nrf2: nuclear factor erythroid 2–related factor 2; PGC-1α: peroxisome proliferator-activated receptor-gamma coactivator 1-alpha; UPRmt: mitochondrial unfolded protein response; GPX: glutathione peroxidases; mtDNA: mitochondrial DNA.

**Table 4:** Stage-specific mitochondria-targeted therapies and limitations in retinal neurodegeneration.

Disease Stage/Mitochondrial State	mtROS Status and QC Capacity	Therapeutic Strategy	Primary Rationale	Key Limitations and Risks	References
Physiological homeostasis	Regulated mtROS Intact QC	No intervention/redox hormesis	Preserves adaptive redox signalling and metabolic flexibility	Risk of unnecessary intervention disrupting physiological signalling	[17,18,19,20]
Compensated mitochondrial stress (Therapeutic window)	Moderately elevated mtROS Stressed but functional QC	Precision mtROS modulation	Restores redox balance without suppressing essential signalling	Narrow therapeutic range Requires accurate disease staging	[19,34,35,36,40]
		QC enhancement (Nrf2 activation, mitophagy support)	Reinforces endogenous stress-adaptation mechanisms	Context- and cell-type–dependent efficacy	[18,20,50,75]
		Mitochondria-targeted antioxidants (e.g., MitoQ, SkQ1)	Reduces excessive mtROS while preserving mitochondrial function	Limited clinical evidence Risk of signalling interference if mistimed	[40,41,42,75,122]
Decompensated mitochondrial stress	High mtROS Failing QC	Combined mtROS attenuation and QC restoration	Slows progression by limiting oxidative amplification	Reduced reversibility Diminished mitochondrial responsiveness	[15,50,75,122,180]
		Modulation of mitochondrial dynamics	Stabilises mitochondrial networks under stress	Off-target effects Potential impairment of adaptive fission–fusion	[50,75,122]
Neurodegeneration (Point of no return)	Self-amplifying mtROS; Collapsed QC	Neuroprotective or symptomatic therapy	Preserves remaining function	Does not restore mitochondrial integrity	[1,7,22,23,24]
		Regenerative/cell-based approaches	Replaces lost or irreversibly damaged cells	Experimental stage Limited integration into existing circuitry	[45,46,180]

mtROS: Mitochondrial reactive oxygen species; QC: Quality control; Nrf2: Nuclear factor erythroid 2–related factor 2; MitoQ: Mitoquinone mesylate; SkQ1: plastoquinonyl-decyltriphenylphosphonium.

## Data Availability

Data sharing is not applicable. No new data were created in this study.

## References

[1] Marchesi N., Fahmideh F., Boschi F., Pascale A., Barbieri A. (2021). Ocular Neurodegenerative Diseases: Interconnection between Retina and Cortical Areas. Cells.

[2] Tham Y.C., Li X., Wong T.Y., Quigley H.A., Aung T., Cheng C.Y. (2014). Global prevalence of glaucoma and projections of glaucoma burden through 2040: A systematic review and meta-analysis. Ophthalmology.

[3] García-García J., Usategui-Martin R., Sanabria M.R., Fernández-Pérez E., Tellería J.J., Coco-Martin R.M. (2022). Pathophysiology of Age-Related Macular Degeneration: Implications for Treatment. Ophthalmic Res..

[4] Teo Z.L., Tham Y.C., Yu M., Chee M.L., Rim T.H., Cheung N., Bikbov M.M., Wang Y.X., Tang Y., Lu Y. (2021). Global Prevalence of Diabetic Retinopathy and Projection of Burden through 2045: Systematic Review and Meta-analysis. Ophthalmology.

[5] Jha R.K., Dawar C., Hasan Q., Pujar A., Gupta G., Vishnu V.Y., Kekunnaya R., Thangaraj K. (2021). Mitochondrial Genetic Heterogeneity in Leber’s Hereditary Optic Neuropathy: Original Study with Meta-Analysis. Genes.

[6] Karaarslan C. (2019). Leber’s Hereditary Optic Neuropathy as a Promising Disease for Gene Therapy Development. Adv. Ther..

[7] Bou Ghanem G.O., Wareham L.K., Calkins D.J. (2024). Addressing neurodegeneration in glaucoma: Mechanisms, challenges, and treatments. Prog. Retin. Eye Res..

[8] Rusciano D., Gagliano C., Avitabile A., Maya-Vetencourt J.F. (2026). Environmental enrichment as a mitochondria-targeting systems strategy across neurodegenerative diseases and retinal dystrophies. Front. Neurosci..

[9] Eells J.T. (2019). Mitochondrial Dysfunction in the Aging Retina. Biology.

[10] Jarrett S.G., Lewin A.S., Boulton M.E. (2010). The Importance of Mitochondria in Age-Related and Inherited Eye Disorders. Ophthalmic Res..

[11] Jarrett S.G., Lewin A.S., Boulton M.E. (2012). The role of mitochondrial oxidative stress in retinal dysfunction. Studies on Retinal and Choroidal Disorders.

[12] Sies H., Jones D.P. (2020). Reactive oxygen species (ROS) as pleiotropic physiological signalling agents. Nat. Rev. Mol. Cell Biol..

[13] Kaarniranta K., Pawlowska E., Szczepańska J., Jablkowska A., Blasiak J. (2019). Role of Mitochondrial DNA Damage in ROS Mediated Pathogenesis of Age-Related Macular Degeneration (AMD). Int. J. Mol. Sci..

[14] Ferrington D.A., Fisher C.R., Kowluru R.A. (2020). Mitochondrial Defects Drive Degenerative Retinal Diseases. Trends Mol. Med..

[15] Rohowetz L.J., Kraus J.G., Koulen P. (2018). Reactive Oxygen Species-Mediated Damage of Retinal Neurons: Drug Development Targets for Therapies of Chronic Neurodegeneration of the Retina. Int. J. Mol. Sci..

[16] Marchi S., Guilbaud E., Tait S.W.G., Yamazaki T., Galluzzi L. (2022). Mitochondrial control of inflammation. Nat. Rev. Immunol..

[17] Mirra S., Marfany G. (2019). Mitochondrial Gymnastics in Retinal Cells: A Resilience Mechanism Against Oxidative Stress and Neurodegeneration. Adv. Exp. Med. Biol..

[18] Yang T.-H., Kang E.Y.-C., Yu B.B.-C., Wang J.H.-H., Chen V., Wang N.-K. (2024). Mitochondria in Retinal Ganglion Cells: Unravelling the Metabolic Nexus and Oxidative Stress. Int. J. Mol. Sci..

[19] Schofield J.H., Schafer Z.T. (2021). Mitochondrial Reactive Oxygen Species and Mitophagy: A Complex and Nuanced Relationship. Antioxid. Redox Signal..

[20] Ye L., Fu X., Li Q. (2025). Mitochondrial Quality Control in Health and Disease. MedComm.

[21] Rusciano D., Bagnoli P. (2024). Oxygen, the paradox of life and the eye. Front. Biosci..

[22] Zhang Z., Zhang X. (2023). Mitochondrial dysfunction in glaucomatous degeneration. Int. J. Ophthalmol..

[23] Johnson L.V., Forest D.L., Banna C.D., Radeke C.M., Maloney M.A., Hu J., Spencer C.N., Walker A.M., Tsie M., Bok D. (2011). Cell culture model that mimics drusen formation and triggers complement activation associated with age-related macular degeneration. Proc. Natl. Acad. Sci. USA.

[24] Somasundaran S., Constable I.J., Mellough C.B., Carvalho L.S. (2020). Retinal pigment epithelium and age-related macular degeneration: A review of major disease mechanisms. Clin. Exp. Ophthalmol..

[25] Wu M.-Y., Yiang G.-T., Lai T.-T., Li C.-J. (2018). The Oxidative Stress and Mitochondrial Dysfunction during the Pathogenesis of Diabetic Retinopathy. Oxidative Med. Cell. Longev..

[26] Alnahdi A.T., Almujalli L.A., Alhawsawi S.Y., Gomawi R.A., Alhobera A.H., Alshammari K.F., Alshammari M., Almuallim H.M., Mubaraki G.K., Shourbaji M.A. (2025). Hyperglycemia-induced oxidative stress in the development of diabetic retinopathy. Int. J. Community Med. Public Health.

[27] Kong G., Van Bergen N.J., Trounce I.A., Crowston J.G. (2009). Mitochondrial dysfunction and glaucoma. J. Glaucoma.

[28] Yu-Wai-Man P., Votruba M., Burté F., La Morgia C., Barboni P., Carelli V. (2016). A neurodegenerative perspective on mitochondrial optic neuropathies. Acta Neuropathol..

[29] Hollyfield J.G. (2010). Age-related macular degeneration: The molecular link between oxidative damage, tissue-specific inflammation and outer retinal disease: The Proctor lecture. Investig. Ophthalmol. Vis. Sci..

[30] de Jong S., Tang J., Clark S.J. (2022). Age-related macular degeneration: A disease of extracellular complement amplification. Immunol. Rev..

[31] Nowak J.Z., Bienias W. (2007). Age-related macular degeneration (AMD): Etiopathogenesis and therapeutic strategies. Postep. Hig. Med. Dosw..

[32] Miller D.J., Cascio M.A., Rosca M.G. (2020). Diabetic Retinopathy: The Role of Mitochondria in the Neural Retina and Microvascular Disease. Antioxidants.

[33] Pan F., Tong J., Wang P., Dong Y. (2024). Research Progress in the Neurovascular Unit Impairment and Treatment of Diabetic Retinopathy. J. Contemp. Med. Pract..

[34] Onukwufor J.O., Berry B.J., Wojtovich A.P. (2019). Physiologic Implications of Reactive Oxygen Species Production by Mitochondrial Complex I Reverse Electron Transport. Antioxidants.

[35] Geto Z., Molla M.D., Challa F., Belay Y., Getahun T. (2020). Mitochondrial Dynamic Dysfunction as a Main Triggering Factor for Inflammation Associated Chronic Non-Communicable Diseases. J. Inflamm. Res..

[36] Li W., Li Y., Zhao J., Liao J., Wen W., Cui H. (2024). Release of Damaged Mitochondrial DNA: A Novel Factor in Stimulating Inflammatory Response. Pathol.-Res. Pract..

[37] Tribble J.R., Otmani A., Sun S., Ellis S.A., Cimaglia G., Vohra R., Jöe M., Lardner E., Venkataraman A.P., Domínguez-Vicent A. (2021). Nicotinamide provides neuroprotection in glaucoma by protecting against mitochondrial and metabolic dysfunction. Redox Biol..

[38] Ren C., Hu C., Wu Y., Li T., Zou A., Yu D., Shen T., Cai W., Yu J. (2022). Nicotinamide Mononucleotide Ameliorates Cellular Senescence and Inflammation Caused by Sodium Iodate in RPE. Oxidative Med. Cell. Longev..

[39] Gemae M.R., Bassi M.D., Wang P., Chin E.K., Almeida D.R.P. (2024). NAD+ and Niacin Supplementation as Possible Treatments for Glaucoma and Age-Related Macular Degeneration: A Narrative Review. Nutrients.

[40] Amorim R., Benfeito S., Teixeira J.A., Cagide F., Oliveira P.J., Borges F., Oliveira P. (2018). Targeting Mitochondria: The Road to Mitochondriotropic Antioxidants and Beyond. Mitochondrial Biology and Experimental Therapeutics.

[41] Ji M.H., Kreymerman A., Belle K., Ghiam B.K., Muscat S., Mahajan V.B., Enns G.M., Mercola M., Wood E.H. (2021). The Present and Future of Mitochondrial-Based Therapeutics for Eye Disease. Transl. Vis. Sci. Technol..

[42] Catalani E., Brunetti K., Del Quondam S., Cervia D. (2023). Targeting Mitochondrial Dysfunction and Oxidative Stress to Prevent the Neurodegeneration of Retinal Ganglion Cells. Antioxidants.

[43] Paß T., Wiesner R.J., Pla-Martín D. (2021). Selective Neuron Vulnerability in Common and Rare Diseases-Mitochondria in the Focus. Front. Mol. Biosci..

[44] Baralić K., Živanović J., Marić Đ., Božić D., Grahovac L., Antonijević Miljaković E., Ćurčić M., Buha Djordjevic A., Bulat Z., Antonijevic B. (2024). Sulforaphane—A Compound with Potential Health Benefits for Disease Prevention and Treatment: Insights from Pharmacological and Toxicological Experimental Studies. Antioxidants.

[45] Palacios A.G., Zhang S.X., Acosta M.L. (2025). Diabetic retinopathy and Alzheimer’s disease: Convergence of the unfolded protein response in neurodegeneration. Alzheimer’s Dement..

[46] Kaštelan S., Kozina L., Alaber M., Tomić Z., Andrešić M., Bakija I., Bućan D., Matejić T., Vidović D. (2025). Neuro-Ophthalmological Disorders Associated with Obstructive Sleep Apnoea. Int. J. Mol. Sci..

[47] Weinreb R.N., Aung T., Medeiros F.A. (2014). The pathophysiology and treatment of glaucoma: A review. JAMA.

[48] Venkatesan A., Bernstein A.M. (2025). Protein misfolding and mitochondrial dysfunction in glaucoma. Front. Cell Dev. Biol..

[49] Hurley D.J., Normile C., Irnaten M., O’Brien C. (2022). The Intertwined Roles of Oxidative Stress and Endoplasmic Reticulum Stress in Glaucoma. Antioxidants.

[50] Kuang G., Halimitabrizi M., Edziah A.-A., Salowe R., O’Brien J.M. (2023). The potential for mitochondrial therapeutics in the treatment of primary open-angle glaucoma: A review. Front. Physiol..

[51] Pietris J. (2022). The Role of NAD+ and Nicotinamide (Vitamin B3) in Glaucoma: A Literature Review. J. Nutr. Sci. Vitaminol..

[52] Stavropoulos D., Grewal M., Petriti B., Chau K., Hammond C.J., Garway-Heath D.F., Lascaratos G. (2023). The Role of Mitophagy in Glaucomatous Neurodegeneration. Cells.

[53] Zhou Z., You M., Fan C., Rong R., Li H., Xia X. (2023). Pathologically high intraocular pressure induces mitochondrial dysfunction through Drp1 and leads to retinal ganglion cell PANoptosis in glaucoma. Redox Biol..

[54] Kaštelan S., Nikuševa-Martić T., Pašalić D., Antunica A.G., Zimak D.M. (2024). Genetic and Epigenetic Biomarkers Linking Alzheimer’s Disease and Age-Related Macular Degeneration. Int. J. Mol. Sci..

[55] Kaštelan S., Tomić M., Gverović Antunica A., Salopek Rabatić J., Ljubić S. (2013). Inflammation and pharmacological treatment in diabetic retinopathy. Mediat. Inflamm..

[56] Kaštelan S., Orešković I., Bišćan F., Kaštelan H., Gverović Antunica A. (2020). Inflammatory and angiogenic biomarkers in diabetic retinopathy. Biochem. Med..

[57] Tomić M., Ljubić S., Kaštelan S. (2013). The role of inflammation and endothelial dysfunction in the pathogenesis of diabetic retinopathy. Coll. Antropol..

[58] Marques-Neves C. (2015). Diabetic retinopathy—Pathophysiology. Acta Ophthalmol..

[59] Sharma I., Yadav K.S., Mugale M.N. (2022). Redoxisome and diabetic retinopathy: Pathophysiology and therapeutic interventions. Pharmacol. Res..

[60] Kowluru R.A., Mohammad G. (2022). Mitochondrial Fragmentation in a High Homocysteine Environment in Diabetic Retinopathy. Antioxidants.

[61] Zhuo Y., Luo H., Zhang K. (2012). Leber hereditary optic neuropathy and oxidative stress. Proc. Natl. Acad. Sci. USA.

[62] Esmaeil A., Ali A., Behbehani R. (2023). Leber’s hereditary optic neuropathy: Update on current diagnosis and treatment. Front. Ophthalmol..

[63] Amore G., Romagnoli M., Carbonelli M., Barboni P., Carelli V., La Morgia C. (2021). Therapeutic Options in Hereditary Optic Neuropathies. Drugs.

[64] Wen H., Deng H., Li B., Chen J., Zhu J., Zhang X., Yoshida S., Zhou Y. (2025). Mitochondrial diseases: From molecular mechanisms to therapeutic advances. Signal Transduct. Target. Ther..

[65] Menger K.E., Logan A., Luhmann U.F.O., Smith A.J., Wright A.F., Ali R.R., Murphy M.P. (2023). In vivo measurement of mitochondrial ROS production in mouse models of photoreceptor degeneration. Redox Biochem. Chem..

[66] King A., Gottlieb E., Brooks D.G., Murphy M.P., Dunaief J.L. (2004). Mitochondria-derived Reactive Oxygen Species Mediate Blue Light-induced Death of Retinal Pigment Epithelial Cells. Photochem. Photobiol..

[67] Barnstable C.J. (2009). Mitochondria and the regulation of free radical damage in the eye. J. Ocul. Biol. Dis. Inform..

[68] Kostyuk A.I., Panova A.S., Kokova A.D., Kotova D.A., Maltsev D.I., Podgorny O.V., Belousov V.V., Bilan D.S. (2020). In Vivo Imaging with Genetically Encoded Redox Biosensors. Int. J. Mol. Sci..

[69] Pedre B. (2024). A guide to genetically-encoded redox biosensors: State of the art and opportunities. Arch. Biochem. Biophys..

[70] Knight L.J., Martis R.M., Donaldson P.J., Acosta M.L., Lim J.C. (2025). Changes in Redox Balance and Mitochondrial Activity in the Retinas of Cystine/Glutamate Antiporter Knockout Mice. Investig. Ophthalmol. Vis. Sci..

[71] Bao Y., Hu C., Wang B., Liu X., Wu Q., Xu D., Shi Z., Sun C. (2025). Mitochondrial Reverse Electron Transport: Mechanisms, Pathophysiological Roles, and Therapeutic Potential. Biology.

[72] Ackermann M.A., Buchholz S.M., Dietrich K., Müller M. (2024). Quantitative, real-time imaging of spreading depolarisation-associated neuronal ROS production. Front. Cell. Neurosci..

[73] Ahsanuddin S., Rios H.A., Otero-Marquez O., Macanian J., Zhou D., Rich C., Rosen R.B. (2023). Flavoprotein fluorescence elevation is a marker of mitochondrial oxidative stress in patients with retinal disease. Front. Ophthalmol..

[74] Arrigo A., Cremona O., Aragona E., Casonic F., Consalezc G., Dogrue R.M., Hauck M.F., Antropolia A., Bianco L., Battaglia Parodia M. (2025). Müller cells trophism and pathology as the next therapeutic targets for retinal diseases. Prog. Retin. Eye Res..

[75] Zhang B., Pan C., Feng C., Yan C., Yu Y., Chen Z., Guo C., Wang X. (2022). Role of mitochondrial reactive oxygen species in homeostasis regulation. Redox Rep..

[76] Böhm E.W., Buonfiglio F., Voigt A.M., Bachmann P., Safi T., Pfeiffer N., Gericke A. (2023). Oxidative stress in the eye and its role in the pathophysiology of ocular diseases. Redox Biol..

[77] Huang S., Van Aken O., Schwarzländer M., Belt K., Millar A.H. (2016). The Roles of Mitochondrial Reactive Oxygen Species in Cellular Signaling and Stress Response in Plants. Plant Physiol..

[78] Kasai S., Shimizu S., Tatara Y., Mimura J., Itoh K. (2020). Regulation of Nrf2 by Mitochondrial Reactive Oxygen Species in Physiology and Pathology. Biomolecules.

[79] Ashrafi G., Schwarz T.L. (2013). The pathways of mitophagy for quality control and clearance of mitochondria. Cell Death Differ..

[80] Calabrese V., Wenzel U., Piccoli T., Jacob U.M., Nicolosi L., Fazzolari G., Failla G., Fritsch T., Osakabe N., Calabrese E.J. (2024). Investigating hormesis, aging, and neurodegeneration: From bench to clinics. Open Med..

[81] Kasai S., Kokubu D., Mizukami H., Itoh K. (2023). Mitochondrial Reactive Oxygen Species, Insulin Resistance, and Nrf2-Mediated Oxidative Stress Response—Toward an Actionable Strategy for Anti-Aging. Biomolecules.

[82] Brooks C.D., Kodati B., Stankowska D.L., Krishnamoorthy R.R. (2023). Role of mitophagy in ocular neurodegeneration. Front. Neurosci..

[83] Yang Y., Zou H. (2025). Research progress on Nrf2 intervention in the treatment of diabetic retinopathy. Front. Endocrinol..

[84] Zhou Q.Y., Ren C., Li J.Y., Wang L., Duan Y., Yao R.Q., Tian Y.P., Yao Y.M. (2024). The crosstalk between mitochondrial quality control and metal-dependent cell death. Cell Death Dis..

[85] Li X., Fang P., Mai J., Choi E.T., Wang H.T., Yang X. (2013). Targeting mitochondrial reactive oxygen species as novel therapy for inflammatory diseases and cancers. J. Hematol. Oncol..

[86] Dabravolski S.A., Nikiforov N.G., Zhuravlev A.D., Orekhov N.A., Grechko A.V., Orekhov A.N. (2022). Role of the mtDNA Mutations and Mitophagy in Inflammaging. Int. J. Mol. Sci..

[87] Li Y., Li X.M., Wei L.S., Ye J.F. (2024). Advancements in mitochondrial-targeted nanotherapeutics: Overcoming biological obstacles and optimizing drug delivery. Front. Immunol..

[88] Al Ojaimi M., Salah A., El-Hattab A.W. (2022). Mitochondrial Fission and Fusion: Molecular Mechanisms, Biological Functions, and Related Disorders. Membranes.

[89] Marchi S., Bittremieux M., Missiroli S., Morganti C., Patergnani S., Sbano L., Rimessi A., Kerkhofs M., Parys J.B., Bultynck G. (2017). Endoplasmic Reticulum-Mitochondria Communication Through Ca^2+^ Signaling: The Importance of Mitochondria-Associated Membranes (MAMs). Adv. Exp. Med. Biol..

[90] Malhotra J.D., Kaufman R.J. (2011). ER Stress and Its Functional Link to Mitochondria: Role in Cell Survival and Death. Cold Spring Harb. Perspect. Biol..

[91] Fan Y., Simmen T. (2019). Mechanistic Connections between Endoplasmic Reticulum (ER) Redox Control and Mitochondrial Metabolism. Cells.

[92] Siegenthaler K.D., Sevier C.S. (2019). Working Together: Redox Signaling between the Endoplasmic Reticulum and Mitochondria. Chem. Res. Toxicol..

[93] Sun Y., Jin L., Qin Y.X., Ouyang Z., Zhong J., Zeng Y. (2024). Harnessing Mitochondrial Stress for Health and Disease: Opportunities and Challenges. Biology.

[94] Kong X., Liu T., Wei J. (2025). Parkinson’s Disease: The Neurodegenerative Enigma Under the “Undercurrent” of Endoplasmic Reticulum Stress. Int. J. Mol. Sci..

[95] Ronayne C.T., Latorre-Muro P. (2024). Navigating the landscape of mitochondrial-ER communication in health and disease. Front. Mol. Biosci..

[96] Wadan A.H.S., Shaaban A.H., El-Sadek M.Z., Mostafa S.A., El-Hussein A., Ellakwa D.E.-S., Mehanny S.S. (2025). Mitochondrial-based therapies for neurodegenerative diseases: A review of the current literature. Naunyn-Schmiedeberg’s Arch. Pharmacol..

[97] Milne G.L., Yin H., Hardy K.D., Davies S.S., Roberts L.J. (2011). Isoprostane generation and function. Chem. Rev..

[98] Tsikas D. (2017). Assessment of lipid peroxidation by measuring malondialdehyde (MDA) and relatives in biological samples: Analytical and biological challenges. Anal. Biochem..

[99] Cilleros-Holgado P., Gómez-Fernández D., Piñero-Pérez R., Romero-Domínguez J.M., Reche-López D., López-Cabrera A., Álvarez-Córdoba M., Munuera-Cabeza M., Talaverón-Rey M., Suárez-Carrillo A. (2023). Mitochondrial Quality Control via Mitochondrial Unfolded Protein Response (mtUPR) in Ageing and Neurodegenerative Diseases. Biomolecules.

[100] Jomova K., Alomar S.Y., Alwasel S.H., Nepovimova E., Kuca K., Valko M. (2024). Several lines of antioxidant defense against oxidative stress: Antioxidant enzymes, nanomaterials with multiple enzyme-mimicking activities, and low-molecular-weight antioxidants. Arch. Toxicol..

[101] Zelko I.N., Mariani T.J., Folz R.J. (2002). Superoxide dismutase multigene family: A comparison of the CuZn-SOD (SOD1), Mn-SOD (SOD2), and EC-SOD (SOD3) gene structures, evolution, and expression. Free Radic. Biol. Med..

[102] Palma F.R., He C., Danes J.M., Paviani V., Coelho D.R., Gantner B.N., Bonini M.G. (2020). Mitochondrial Superoxide Dismutase: What the Established, the Intriguing, and the Novel Reveal About a Key Cellular Redox Switch. Antioxid. Redox Signal..

[103] Pei J., Pan X., Wei G., Hua Y. (2023). Research progress of glutathione peroxidase family (GPX) in redoxidation. Front. Pharmacol..

[104] Stolwijk J.M., Falls-Hubert K.C., Searby C.C., Wagner B.A., Buettner G.R. (2020). Simultaneous detection of the enzyme activities of GPx1 and GPx4 guide optimization of selenium in cell biological experiments. Redox Biol..

[105] Domènech B.E., Marfany G. (2020). The Relevance of Oxidative Stress in the Pathogenesis and Therapy of Retinal Dystrophies. Antioxidants.

[106] Sreekumar P.G., Ferrington D.A., Kannan R. (2021). Glutathione Metabolism and the Novel Role of Mitochondrial GSH in Retinal Degeneration. Antioxidants.

[107] Lu L., Oveson B.C., Jo Y.J., Lauer T.W., Usui S., Komeima K., Xie B., Campochiaro P.A. (2009). Increased expression of glutathione peroxidase 4 strongly protects retina from oxidative damage. Antioxid. Redox Signal..

[108] Nandi A., Yan L.J., Jana C.K., Das N. (2019). Role of Catalase in Oxidative Stress- and Age-Associated Degenerative Diseases. Oxidative Med. Cell. Longev..

[109] Baker A., Lin C.C., Lett C., Karpinska B., Wright M.H., Foyer C.H. (2023). Catalase: A critical node in the regulation of cell fate. Free Radic. Biol. Med..

[110] Mimura T., Noma H. (2025). Oxidative Stress in Age-Related Macular Degeneration: From Molecular Mechanisms to Emerging Therapeutic Targets. Antioxidants.

[111] Kameritsch P., Singer M., Nuernbergk C., Rios N., Reyes A.M., Schmidt K., Kirsch J., Schneider H., Müller S., Pogoda K. (2021). The mitochondrial thioredoxin reductase system (TrxR2) in vascular endothelium controls peroxynitrite levels and tissue integrity. Proc. Natl. Acad. Sci. USA.

[112] Ngo V., Duennwald M.L. (2022). Nrf2 and Oxidative Stress: A General Overview of Mechanisms and Implications in Human Disease. Antioxidants.

[113] Saso L., Ates I., Tunc R., Yilmaz B., Gallorini M., Carradori S., Suzen S. (2025). Modulation of Nrf2 and mitochondrial function. Pharmaceuticals.

[114] Hu Z.L., Wang Y.X., Lin Z.Y., Ren W.S., Liu B., Zhao H., Qin Q. (2024). Regulatory factors of Nrf2 in age-related macular degeneration pathogenesis. Int. J. Ophthalmol..

[115] Brown E.E., DeWeerd A.J., Ildefonso C.J., Lewin A.S., Ash J.D. (2019). Mitochondrial oxidative stress in the retinal pigment epithelium (RPE) led to metabolic dysfunction in both the RPE and retinal photoreceptors. Redox Biol..

[116] Ichimura Y., Waguri S., Sou Y.S., Kageyama S., Hasegawa J., Ishimura R., Saito T., Yang Y., Kouno T., Fukutomi T. (2013). Phosphorylation of p62 activates the Keap1-Nrf2 pathway during selective autophagy. Mol. Cell.

[117] Zanfardino P., Amati A., Perrone M., Petruzzella V. (2025). The Balance of MFN2 and OPA1 in Mitochondrial Dynamics, Cellular Homeostasis, and Disease. Biomolecules.

[118] Chen W., Zhao H., Li Y. (2023). Mitochondrial dynamics in health and disease: Mechanisms and potential targets. Signal Transduct. Target. Ther..

[119] Vidoni S., Zanna C., Rugolo M., Sarzi E., Lenaers G. (2013). Why mitochondria must fuse to maintain genome integrity. Antioxid. Redox Signal..

[120] Zerihun M., Sukumaran S., Qvit N. (2023). The Drp1-Mediated Mitochondrial Fission Protein Interactome as an Emerging Core Player in Mitochondrial Dynamics and Cardiovascular Disease Therapy. Int. J. Mol. Sci..

[121] Twig G., Elorza A., Molina A.J., Mohamed H., Wikstrom J.D., Walzer G., Stiles L., Haigh S.E., Katz S., Las G. (2008). Fission and selective fusion govern mitochondrial segregation. EMBO J..

[122] Ježek J., Cooper K.F., Strich R. (2018). Reactive Oxygen Species and Mitochondrial Dynamics. Antioxidants.

[123] Jin S.M., Youle R.J. (2013). The accumulation of misfolded proteins in the mitochondrial matrix is sensed by PINK1 to induce PARK2/Parkin-mediated mitophagy of polarized mitochondria. Autophagy.

[124] Quinn P.M.J., Moreira P.I., Ambrósio A.F., Alves C.H. (2020). PINK1/PARKIN signalling in neurodegeneration and neuroinflammation. Acta Neuropathol. Commun..

[125] Onishi M., Yamano K., Sato M., Matsuda N., Okamoto K. (2021). Mechanisms and physiological functions of mitophagy. EMBO J..

[126] Abu Shelbayeh O., Arroum T., Morris S., Busch K.B. (2023). PGC-1α Is a Master Regulator of Mitochondrial Lifecycle and ROS Stress Response. Antioxidants.

[127] MacKenzie J.A., Payne R.M. (2007). Mitochondrial protein import and human health. Biochim. Biophys. Acta (BBA)-Mol. Basis Dis..

[128] Naresh N.U., Haynes C.M. (2019). Signaling and Regulation of the Mitochondrial UPR. Cold Spring Harb. Perspect. Biol..

[129] Lu H., Wang X., Li M., Ji D., Liang D., Liang C., Liu Y., Zhang Z., Cao Y., Zou W. (2022). Mitochondrial Unfolded Protein Response and Integrated Stress Response as Promising Therapeutic Targets for Mitochondrial Diseases. Cells.

[130] Zhang X., Fan Y., Tan K. (2024). A bird’s-eye view of mitochondrial UPR in cancer. Cell Death Dis..

[131] Teske B.F., Fusakio M.E., Zhou D., Shan J., McClintick J.N., Kilberg M.S., Wek R.C. (2013). CHOP induces activating transcription factor 5 (ATF5) to trigger apoptosis in response to perturbations in protein homeostasis. Mol. Biol. Cell.

[132] Iside C., Scafuro M., Nebbioso A., Altucci L. (2020). SIRT1 Activation by Natural Phytochemicals: An Overview. Front. Pharmacol..

[133] Xu G., Gu Y., Yan N., Li Y., Sun L., Li B. (2021). Curcumin functions as an anti-inflammatory and antioxidant agent on arsenic-induced hepatic and kidney injury by inhibiting MAPKs/NF-κB and activating Nrf2 pathways. Environ. Toxicol..

[134] Di Nicolantonio J.J., McCarty M.F., O’Keefe J.H. (2022). Nutraceutical activation of Sirt1: A review. Open Heart.

[135] Kaštelan S., Konjevoda S., Sarić A., Urlić I., Lovrić I., Čanović S., Matejić T., Šešelja Perišin A. (2025). Resveratrol as a Novel Therapeutic Approach for Diabetic Retinopathy. Molecules.

[136] Khan R.S., Fonseca-Kelly Z., Callinan C.E., Zuo L., Sachdeva M.M., Shindler K.S. (2012). SIRT1 activating compounds reduce oxidative stress and prevent cell death in neuronal cells. Front. Cell. Neurosci..

[137] Stice S.A., Kolanos R. (2021). Nutraceuticals in ophthalmic diseases. Nutraceuticals.

[138] Sun G.-F., Qu X., Jiang L.-P., Chen Z.-P., Han X.-J. (2024). The mechanisms of natural products for eye disorders by targeting mitochondrial dysfunction. Front. Pharmacol..

[139] Kubota S., Kurihara T., Mochimaru H., Satofuka S., Noda K., Ozawa Y., Oike Y., Ishida S., Tsubota K. (2009). Prevention of Ocular Inflammation in Endotoxin-Induced Uveitis with Resveratrol by Inhibiting Oxidative Damage and Nuclear Factor-κB Activation. Investig. Ophthalmol. Vis. Sci..

[140] Chen J., Zhang W., Zheng Y., Xu Y. (2022). Ameliorative Potential of Resveratrol in Dry Eye Disease by Restoring Mitochondrial Function. Evid.-Based Complement. Altern. Med..

[141] Delmas D., Cornebise C., Courtaut F., Xiao J., Aires V. (2021). New Highlights of Resveratrol: A Review of Properties against Ocular Diseases. Int. J. Mol. Sci..

[142] Rahban M., Habibi-Rezaei M., Mazaheri M., Saso L., Moosavi-Movahedi A.A. (2020). Anti-Viral Potential and Modulation of Nrf2 by Curcumin: Pharmacological Implications. Antioxidants.

[143] Ashrafizadeh M., Ahmadi Z., Mohammadinejad R., Farkhondeh T., Samarghandian S. (2020). Curcumin Activates the Nrf2 Pathway and Induces Cellular Protection Against Oxidative Injury. Curr. Mol. Med..

[144] Alrawaiq N.S., Abdullah A. (2014). A review of antioxidant polyphenol curcumin and its role in detoxification. Int. J. PharmTech Res..

[145] Lee H., Kim S.W., Lee H.-K., Luo L., Kim I.-D., Lee J.-K. (2016). Upregulation of Nrf2–p300 mediates anti-inflammatory effects of curcumin in microglia by downregulating p65–p300. Anim. Cells Syst..

[146] Castro-Castaneda C.R., Altamirano-Lamarque F., Ortega-Macías A.G., Santa Cruz-Pavlovich F.J., Gonzalez-De la Rosa A., Armendáriz-Borunda J., Santos A., Navarro-Partida J. (2022). Nutraceuticals: A Promising Therapeutic Approach in Ophthalmology. Nutrients.

[147] Li D., Shao R., Wang N., Zhou N., Du K., Shi J., Wang Y., Zhao Z., Ye X., Zhang X. (2020). Sulforaphane Activates a Lysosome-dependent Transcriptional Program to Mitigate Oxidative Stress. Autophagy.

[148] Luján L.M.L., McCarty M.F., Di Nicolantonio J.J., Gálvez Ruiz J.C., Rosas-Burgos E.C., Plascencia-Jatomea M., Iloki Assanga S.B. (2022). Nutraceuticals/Drugs Promoting Mitophagy and Mitochondrial Biogenesis May Combat the Mitochondrial Dysfunction Driving Progression of Dry Age-Related Macular Degeneration. Nutrients.

[149] Zhang Y., Zhao X., Liu Y., Yang X. (2024). Sulforaphane and ophthalmic diseases. Food Sci. Nutr..

[150] Hui F., Tang J., Williams P.A., McGuinness M.B., Hadoux X., Casson R.J., Coote M., Trounce I.A., Martin K.R., van Wijngaarden P. (2020). Improvement in inner retinal function in glaucoma with nicotinamide (vitamin B3) supplementation: A crossover randomized clinical trial. Clin. Exp. Ophthalmol..

[151] Cimaglia G., Tribble J.R., Votruba M., Williams P.A., Morgan J.S. (2024). Oral nicotinamide provides robust, dose-dependent structural and metabolic neuroprotection of retinal ganglion cells in experimental glaucoma. Acta Neuropathol. Commun..

[152] Yi B., Zeng J., Li J., Liu K., Zhu X., Chen X., Gao Y. (2025). Idebenone: Clinical Potential Beyond Neurological Diseases. Drug Des. Dev. Ther..

[153] Pickles S., Vigié P., Youle R.J. (2018). Mitophagy and Quality Control Mechanisms in Mitochondrial Maintenance. Curr. Biol..

[154] Telegina D.V., Kozhevnikova O.S., Fursova A.Z., Kolosova N.G. (2020). Autophagy as a Target for the Retinoprotective Effects of the Mitochondria-Targeted Antioxidant SkQ1. Biochemistry.

[155] Huang B., Zhang N., Qiu X., Zeng R., Wang S., Hua M., Li Q., Nan K., Lin S. (2023). Mitochondria-targeted SkQ1 nanoparticles for dry eye disease: Inhibiting NLRP3 inflammasome activation by preventing mitochondrial DNA oxidation. J. Control. Release.

[156] Feniouk B.A., Skulachev M.V. (2018). SkQ1: The Road from Laboratory Bench to the Market. Mitochondrial Biology and Experimental Therapeutics.

[157] Muraleva N.A., Zhdankina A.A., Fursova A.Z., Kolosova N.G. (2024). Retinoprotective Effect of SkQ1, Visomitin Eye Drops, Is Associated with Suppression of P38 MAPK and ERK1/2 Signaling Pathways Activity. Biochemistry.

[158] Tung C., Varzideh F., Farroni E., Mone P., Kansakar U., Jankauskas S.S., Santulli G. (2025). Elamipretide: A Review of Its Structure, Mechanism of Action, and Therapeutic Potential. Int. J. Mol. Sci..

[159] Pang Y., Wang C., Yu L. (2015). Mitochondria-Targeted Antioxidant SS-31 is a Potential Novel Ophthalmic Medication for Neuroprotection in Glaucoma. Med. Hypothesis Discov. Innov. Ophthalmol..

[160] Du X., Zeng Q., Luo Y., He L., Zhao Y., Li N., Han C., Zhang G., Liu W. (2024). Application Research of Novel Peptide Mitochondrial-Targeted Antioxidant SS-31 in mitigating Mitochondrial Dysfunction. Mitochondrion.

[161] Ehlers J.P., Hu A., Boyer D., Cousins S.W., Waheed N.K., Rosenfeld P.J., Brown D., Kaiser P.K., Abbruscato A., Gao G. (2024). ReCLAIM-2: A Randomized Phase II Clinical Trial Evaluating Elamipretide in Age-related Macular Degeneration, Geographic Atrophy Growth, Visual Function, and Ellipsoid Zone Preservation. Ophthalmol. Sci..

[162] Chiu T.H., Hung S.H., Lan C.H., Yen W.T., Lu D.W. (2025). Update on Nicotinamide and Its Application in the Management of Glaucoma. Int. J. Mol. Sci..

[163] Grosso A., Borrelli E., Sacchi M., Sacchi M., Calzetti G., Ceruti P., Neri G., Marchetti M., Pinna A., Kostin V. (2025). Neuroprotection beyond intraocular pressure: Game changer or quiet addiction. Graefe’s Arch. Clin. Exp. Ophthalmol..

[164] Yusri K., Jose S., Vermeulen K.S., Tan T.C.M., Sorrentino V. (2025). The role of NAD^+^ metabolism and its modulation of mitochondria in aging and disease. NPJ Metab. Health Dis..

[165] Nhan N.T.T., Maidana D.E., Yamada K.H. (2023). Ocular Delivery of Therapeutic Agents by Cell-Penetrating Peptides. Cells.

[166] Chernega T., Choi J., Salmena L., Andreazza A.C. (2022). Mitochondrion-targeted RNA therapies as a potential treatment strategy for mitochondrial diseases. Mol. Ther. Nucleic Acids.

[167] Skeie J.M., Nishimura D.Y., Wang C.L., Schmidt G.A., Aldrich B.T., Greiner M.A. (2021). Mitophagy: An Emerging Target in Ocular Pathology. Investig. Ophthalmol. Vis. Sci..

[168] Huang S., Liu C.H., Wang Z., Fu Z., Britton W.R., Blomfield A.K., Kamenecka T.M., Dunaief J.L., Solt L.A., Chen J. (2022). REV-ERBα regulates age-related and oxidative stress-induced degeneration in retinal pigment epithelium via NRF2. Redox Biol..

[169] Zhang T., Liu Q., Gao W., Sehgal S.A., Wu H. (2022). The multifaceted regulation of mitophagy by endogenous metabolites. Autophagy.

[170] Held N.M., Houtkooper R.H. (2015). Mitochondrial quality control pathways as determinants of metabolic health. Bioessays.

[171] Liu B.H., Xu C.Z., Liu Y., Lu Z.L., Fu T.L., Li G.R., Deng Y., Luo G.Q., Ding S., Li N. (2024). Mitochondrial quality control in human health and disease. Mil. Med. Res..

[172] Zorova L.D., Popkov V.A., Plotnikov E.Y., Silachev D.N., Pevzner I.B., Jankauskas S.S., Babenko V.A., Zorov S.D., Balakireva A.V., Juhaszova M. (2018). Mitochondrial membrane potential. Anal. Biochem..

[173] Zhang S.M., Fan B., Li Y.L., Zuo Z.Y., Li G.Y. (2023). Oxidative Stress-Involved Mitophagy of Retinal Pigment Epithelium and Retinal Degenerative Diseases. Cell. Mol. Neurobiol..

[174] Cheng Y., Cai S., Wu H., Pan J., Su M., Wei X., Ye J., Ke L., Liu G., Chu C. (2024). Revolutionizing eye care: The game-changing applications of nano-antioxidants in ophthalmology. Nanoscale.

[175] Rowe L.W., Akotoye C., Harris A., Ciulla T.A. (2025). Beyond the injection: Delivery systems reshaping retinal disease management. Expert Opin. Pharmacother..

[176] Sánchez-López E., Espina M., Doktorovova S., Souto E.B., García M.L. (2017). Lipid nanoparticles in ocular delivery. Eur. J. Pharm. Biopharm..

[177] Gaudana R., Ananthula H.K., Parenky A., Mitra A.K. (2010). Ocular drug delivery. AAPS J..

[178] Fields M., Marcuzzi A., Gonelli A., Celeghini C., Maximova N., Rimondi E. (2023). Mitochondria-Targeted Antioxidants, an Innovative Class of Antioxidant Compounds for Neurodegenerative Diseases: Perspectives and Limitations. Int. J. Mol. Sci..

[179] Chrysostomou V., Rezania F., Trounce I.A., Crowston J.G. (2013). Oxidative stress and mitochondrial dysfunction in glaucoma. Curr. Opin. Pharmacol..

[180] Jumelle C., Gholizadeh S., Annabi N., Dana R. (2020). Advances and limitations of drug delivery systems formulated as eyedrops. J. Control. Release.

[181] Wang J., Li M., Geng Z., Khattak S., Ji X., Wu D., Dang Y. (2022). Oxidative Stress in Retinal Disease. Oxidative Med. Cell. Longev..

[182] Tawfik M., Chen F., Goldberg J.L., Sabel B.A. (2022). Nanomedicine and drug delivery to the retina. Naunyn-Schmiedeberg’s Arch. Pharmacol..

[183] Patel M.J., Sheth S., Mar J., Gregori N.Z., Sengillo J.D. (2025). Surgical Approaches to Retinal Gene Therapy. Bioengineering.

[184] Wu K.Y., Fujioka J.K., Gholamian T., Zaharia M., Tran S.D. (2023). Suprachoroidal Injection: A Novel Approach for Targeted Drug Delivery. Pharmaceuticals.

[185] Mishra G.P., Bagui M., Tamboli V., Mitra A.K. (2011). Application of liposomes in ophthalmic drug delivery. J. Drug Deliv..

[186] Xue B., Ge M., Fan K., Huang X., Yan X., Jiang W., Jiang B., Yang Z. (2022). Mitochondria-targeted nanozyme in retinal neovascularisation. J. Control. Release.

[187] Alshaikh R.A., Waeber C., Ryan K.B. (2022). Polymer based sustained drug delivery to the ocular posterior segment: Barriers and future opportunities for the treatment of neovascular pathologies. Adv. Drug Deliv. Rev..

[188] Omidian H., Wilson R.L. (2025). PLGA Implants for Controlled Drug Delivery. Pharmaceuticals.

[189] Batheja S., Gupta S., Tejavath K.K., Gupta U. (2024). TPP-based conjugates: Potential targeting ligands. Drug Discov. Today.

[190] Nahar N., Sohag M.S.U. (2025). Advancements in mitochondrial-targeted antioxidants. Arch. Res. Med..

[191] Rodríguez Villanueva J., Martín Esteban J., Rodríguez Villanueva L.J. (2020). Retinal cell protection & microparticulate delivery. Pharmaceutics.

[192] Kishore K., Bhat P.V., Venkatesh P., Canizela C.C. (2022). Dexamethasone Intravitreal Implant in Macular Oedema and Uveitis. Clin. Ophthalmol..

[193] Parvin N., Joo S.W., Mandal T.K. (2025). Biodegradable and stimuli-responsive nanomaterials. J. Funct. Biomater..

[194] Mashele S.S. (2025). Stimuli-responsive, cell-mediated drug delivery systems. Pharmaceutics.

[195] Imtiaz S., Ferdous U.T., Nizela A., Hasan A., Shakoor A., Zia A.W., Uddin S. (2025). Mechanistic study of cancer drug delivery: Current techniques, limitations, and future prospects. Eur. J. Med. Chem..

[196] Jensen C., Teng Y. (2020). Is It Time to Start Transitioning From 2D to 3D Cell Culture?. Front. Mol. Biosci..

[197] Heller J.P., Kwok J.C., Vecino E., Martin K.R., Fawcett J.W. (2015). A Method for the Isolation and Culture of Adult Rat Retinal Pigment Epithelial (RPE) Cells to Study Retinal Diseases. Front. Cell. Neurosci..

[198] Kozlowski M.R. (2015). The ARPE-19 cell line: Mortality status and utility in macular degeneration research. Curr. Eye Res..

[199] Blenkinsop T.A., Salero E., Stern J.H., Temple S. (2013). The culture and maintenance of functional retinal pigment epithelial monolayers from adult human eye. Methods Mol. Biol..

[200] Zhao H., Yan F. (2024). Retinal Organoids: A Next-Generation Platform for High-Throughput Drug Discovery. Stem Cell Rev. Rep..

[201] Qamar F., Sultana S., Sharma M. (2023). Animal models for induction of diabetes and its complications. J. Diabetes Metab. Disord..

[202] Pennesi M.E., Neuringer M., Courtney R.J. (2012). Animal models of age related macular degeneration. Mol. Asp. Med..

[203] Alfonsetti M., Castelli V., Angelo M., Benedetti E., Allegretti M., Barboni B., Cimini A. (2021). Looking for In Vitro Models for Retinal Diseases. Int. J. Mol. Sci..

[204] Diot A., Hinks-Roberts A., Lodge T., Liao C., Dombi E., Morten K., Brady S., Fratter C., Carver J., Muir R. (2015). A novel quantitative assay of mitophagy: Combining high content fluorescence microscopy and mitochondrial DNA load to quantify mitophagy and identify novel pharmacological tools against pathogenic heteroplasmic mtDNA. Pharmacol. Res..

[205] Primiano G., Abed E., Corbo G., Minnella A.M., Servidei S., Vollono C., Savastano M.C., Falsini B. (2020). Macular impairment in mitochondrial diseases: A potential biomarker of disease severity. Sci. Rep..

[206] Cao D., Leong B., Messinger J.D., Kar D., Ach T., Yannuzzi L.A., Freund K.B., Curcio C.A. (2021). Hyperreflective Foci, Optical Coherence Tomography Progression Indicators in Age-Related Macular Degeneration, Include Transdifferentiated Retinal Pigment Epithelium. Investig. Ophthalmol. Vis. Sci..

[207] Kaštelan S., Gverović Antunica A., Puzović V., Didović Pavičić A., Ćanović S., Kovačević P., Vučemilović P.A.F., Konjevoda S. (2025). Non-Invasive Retinal Biomarkers for Early Diagnosis of Alzheimer’s Disease. Biomedicines.

[208] Karunadharma P.P., Nordgaard C.L., Olsen T.W., Ferrington D.A. (2010). Mitochondrial DNA damage as a potential mechanism for age-related macular degeneration. Investig. Ophthalmol. Vis. Sci..

[209] Chen A.X., Conti T.F., Hom G.L., Greenlee T., Raimondi R., Briskin I.N., Rich C.A., Kampani R., Engel R., Sharma S. (2021). Functional imaging of mitochondria in retinal diseases using flavoprotein fluorescence. Eye.

[210] Carrella S., Massa F., Indrieri A. (2021). The Role of MicroRNAs in Mitochondria-Mediated Eye Diseases. Front. Cell Dev. Biol..

[211] Nogueira-Machado J.A., da Silva Albanaz A.T., Rocha-Silva F. (2025). MicroRNA as a Potential Biomarker for Amyotrophic Lateral Sclerosis (ALS). Sclerosis.

[212] Paramasivam A., Priyadharsini J.V. (2020). MitomiRs: New emerging microRNAs in mitochondrial dysfunction and cardiovascular disease. Hypertens. Res..

[213] Yu-Wai-Man P., Newman N.J., Biousse V., Carelli V., Moster M.L., Vignal-Clermont C., Klopstock T., Sadun A.A., Sergott R.C., Hage R. (2025). A Five-Year Outcomes of Lenadogene Nolparvovec Gene Therapy in Leber Hereditary Optic Neuropathy. JAMA Ophthalmol..

[214] Zinovkin R.A., Zamyatnin A.A. (2019). Mitochondria-Targeted Drugs. Curr. Mol. Pharmacol..

[215] Davì F., Iaconis A., Cordaro M., Di Paola R., Fusco R. (2025). Nutraceutical Strategies for Targeting Mitochondrial Dysfunction in Neurodegenerative Diseases. Foods.

[216] Baglivo M., Nasca A., Lamantea E., Vinci S., Spagnolo M., Marchet S., Prokisch H., Catania A., Lamperti C., Ghezzi D. (2023). Evaluation of Mitochondrial Dysfunction and Idebenone Responsiveness in Fibroblasts from Leber’s Hereditary Optic Neuropathy (LHON) Subjects. Int. J. Mol. Sci..

[217] Barot M., Gokulgandhi M.R., Mitra A.K. (2011). Mitochondrial dysfunction in retinal diseases. Curr. Eye Res..

[218] Carley A.N., Lewandowski E.D. (2014). Is the Therapeutic Window for Mitochondrial ROS Half-Open or Half-Closed?: Mixing Mitophagic Metaphors. Circ. Res..

[219] Ghosh D., Kumar A. (2024). Harnessing Mitophagy for Therapeutic Advances in Aging and Chronic Neurodegenerative Diseases. Neuroglia.

[220] Dhar K.L., Townsend B., Montgomery A.P., Danon J.J., Pagan J.K., Kassiou M. (2024). Enhancing CNS mitophagy: Drug development and disease-relevant models. Trends Pharmacol. Sci..

[221] Ferro F., Servais S., Besson P., Roger S., Dumas J.F., Brisson L. (2020). Autophagy and mitophagy in cancer metabolic remodelling. Semin. Cell Dev. Biol..

[222] Zhang H., Xie S., Deng W. (2024). Mitophagy in Doxorubicin-Induced Cardiotoxicity: Insights into Molecular Biology and Novel Therapeutic Strategies. Biomolecules.

